# Generalized spatial mark–resight models with incomplete identification: An application to red fox density estimates

**DOI:** 10.1002/ece3.5077

**Published:** 2019-03-22

**Authors:** Jose Jimenez, Richard Chandler, Jorge Tobajas, Esther Descalzo, Rafael Mateo, Pablo Ferreras

**Affiliations:** ^1^ Instituto de Investigación en Recursos Cinegéticos (IREC, CSIC‐UCLM‐JCCM) Ciudad Real Spain; ^2^ Warnell School of Forestry and Natural Resources University of Georgia Athens Georgia

**Keywords:** camera trapping, generalized spatial mark–resight, incomplete identification, mark, red fox, telemetry

## Abstract

1. The estimation of abundance of wildlife populations is an essential part of ecological research and monitoring. Spatially explicit capture–recapture (SCR) models are widely used for abundance and density estimation, frequently through individual identification of target species using camera‐trap sampling.
2. Generalized spatial mark–resight (Gen‐SMR) is a recently developed SCR extension that allows for abundance estimation when only a subset of the population is recognizable by artificial or natural marks. However, in many cases, it is not possible to read the marks in camera‐trap pictures, even though individuals can be recognized as marked. We present a new extension of Gen‐SMR that allows for this type of incomplete identification.
3. We used simulation to assess how the number of marked individuals and the individual identification rate influenced bias and precision. We demonstrate the model's performance in estimating red fox (*Vulpes vulpes*) density with two empirical datasets characterized by contrasting densities and rates of identification of marked individuals. According to the simulations, accuracy increases with the number of marked individuals (*m*), but is less sensitive to changes in individual identification rate (δ). In our case studies of red fox density estimation, we obtained a posterior mean of 1.60 (standard deviation SD: 0.32) and 0.28 (*SD*: 0.06) individuals/km^2^, in high and low density, with an identification rate of 0.21 and 0.91, respectively.
4. This extension of Gen‐SMR is broadly applicable as it addresses the common problem of incomplete identification of marked individuals during resighting surveys.

## 1 INTRODUCTION

Capture–recapture (CR) methods are considered reference methods in population size estimates (Silvy, 2012). They use the capture histories of animals with natural traits or artificial markings, extending inferences from the detected individuals to the population size. However, some of the assumptions, such as homogeneity in capture probability of individuals are violated by the implicit heterogeneity derived from the location of the activity center of each animal in relation to each trap or detection device. Another limitation of standard CR methods is that they cannot be used for density estimation because the effective sampling area is unknown (Otis, Burnham, White, & Anderson, 1978; Karanth & Nichols, 1998; Parmenter et al., 2003; Soisalo & Cavalcanti, 2006). These disadvantages of CR are overcome with spatially explicit capture–recapture (SCR) methods, which have been recently developed (Borchers & Efford, 2008; Efford, Dawson, & Robbins, 2004; Kéry, Gardner, Stoeckle, Weber, & Royle, 2011; Royle, Chandler, Sollmann, & Gardner, 2014; Royle & Young, 2008).

Spatially explicit capture–recapture models are thinned spatial point process models used to make inferences about the abundance and distribution of animal activity centers (Efford et al., 2004; Royle et al. 2014). SCR models allow for inference about individual heterogeneity by modeling capture probability as a function of the distance between activity centers and detectors. The SCR capture probability function typically includes two main parameters: the scale parameter of the half‐normal distribution (*σ*), which is determined by home range size; and the baseline detection rate (*λ*_0_), that is the probability of encountering an individual at its activity center. The probability of detection of an individual in a detector depends on the Euclidean distance between its center of activity and the detector location, *σ* and *λ*_0_. Despite its utility in studies of marked animals, SCR applicability is limited in studies that yield data on unmarked or partially populations. For example, in camera‐traps studies, the majority of detected species typically do not have individually recognizable natural or artificial marks, making it impossible to develop the capture histories required by SCR models.

Spatial mark–resight (SMR) is an extension of SCR that can be used when only a fraction of the animals can be uniquely identified. SMR methods use the encounter histories from the marked portion of the population (by artificial or natural marks) and the counts of unmarked individuals to estimate density and detection parameters (Chandler & Royle, 2013; Sollmann, Gardner et al., 2013). If some individuals require artificial marks for identification, SMR methods require a live‐trapping period for tagging, and a subsequent sampling period to collect capture histories of both tagged and unmarked individuals (Jiménez, Higuero, Charre‐Medellín, & Acevedo, 2017; Royle et al., 2014; Sollmann, Gardner et al., 2013). Sollmann, Gardner et al. (2013) proposed the additional use of telemetry data to enhance estimation of the detection parameters when the number of spatial recaptures (captures of the same animal in different traps) is insufficient. Using telemetry is common in field studies, and this extension of SMR facilitated its application to the estimates. However, an unsolved problem remained: the requirement that the marked animals should be a random subset of the population (Royle et al., 2014). This requirement was overcome with advent of generalized SMR (Gen‐SMR) models (Efford & Hunter, 2018; Whittington, Hebblewhite, & Chandler, 2017) which include sub‐models for both marking and resighting processes. The model for the marking process describes the distribution of the marked individual, thereby relaxing the SMR assumption that marked and unmarked populations have the same spatial distributions and encounter probabilities (Whittington et al., 2017).

A very common problem in mark–resight studies is the difficulty of identifying marked individuals with certainty during resighting surveys (McClintock et al., 2014). Incomplete marked individual identification cannot typically be eliminated by study design alone (e.g., using more than one camera‐trap in each point, or using more than one tag in each individual).

McClintock et al. (2014) used a method that accounts for uncertainty in marked individual detection histories when incomplete identifications occur for the two approaches commonly used in nonspatial mark–resight models of abundance, the Poisson log‐normal estimator and the logit‐normal estimator. MCMC approaches to deal with this issue could be found in Whittington et al. (2017) and Augustine, Royle, Stewart, Fisher, and Kelly (2018), and using MLE in Efford and Hunter (2018).

Here, we present a Gen‐SMR extension for a known number of marked individuals that accounts for incomplete individual identification and makes full use of spatial information.

## 2 MATERIALS AND METHODS

### 2.1 Model

**Figure 1:**
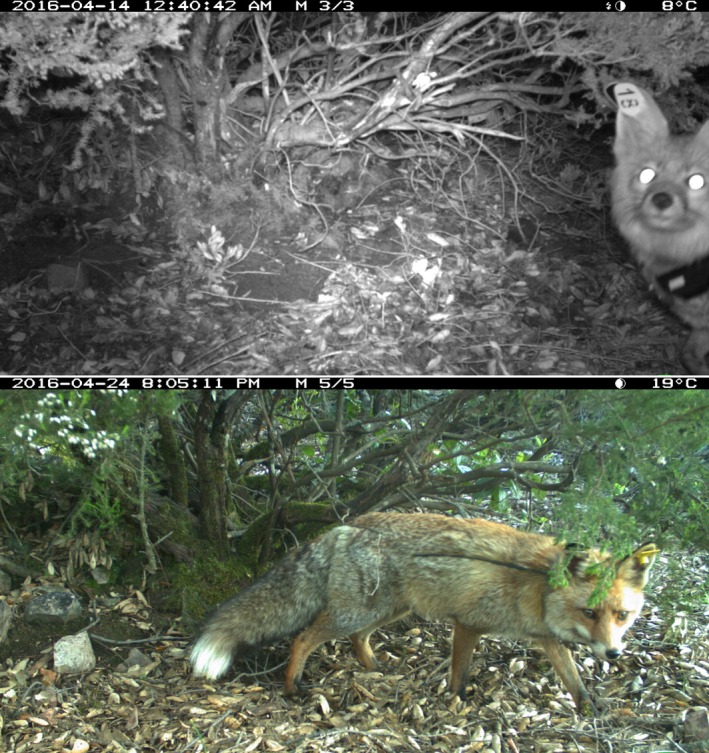
Two typical camera‐trap pictures of marked individuals. On top, picture with an individual identified by the mark number on the right ear. Bottom: none of the tags are visible, and the individual is unidentifiable. Photo credit: authors

The partial identification extension that we propose for generalized spatial mark–resight (hereafter Gen‐SMR‐ID) is based on the (Whittington et al., 2017) model that included marking and resighting processes in the model, as well as the integration of telemetry data. Gen‐SMR‐ID is designed to deal with incomplete marked individual identification during resighting surveys in SMR approaches. Our extension can be used when the number of marked individuals is known, and when, in the resighting process, a portion of or all marked individuals cannot be identified (Figure 1).

#### 2.1.1 Ecological process

We used the same underlying ecological and marking process as described by Whittington et al. (2017). As with standard SCR applications, we model a spatial variation in density using as a spatial point process model with *N* latent activity centers *s_1_,s_2_,…,s_N_* in the state space (*S*). If density can be assumed to be constant across S, the activity centers follow a uniform distribution, *s_i_ ~ *Unif(*S*).

#### 2.1.2 Marking process

Let *x_j_*be the Cartesian coordinates for trap *j* (*j = 1, …, J*). The encounter function depends on the Euclidean distance between traps and activity centers, $d_{\mathrm{ij}}^{N}=s_{i}-x_{j}$, the scale parameter sigma (*σ*) and the baseline detection (here, capture) rate (*λ*_0.mark_). The marking process can be modeled using a binomial, Poisson, or categorical distribution, depending on the detector devices used in this model.

#### 2.1.3 Resighting process

**Figure 2:**
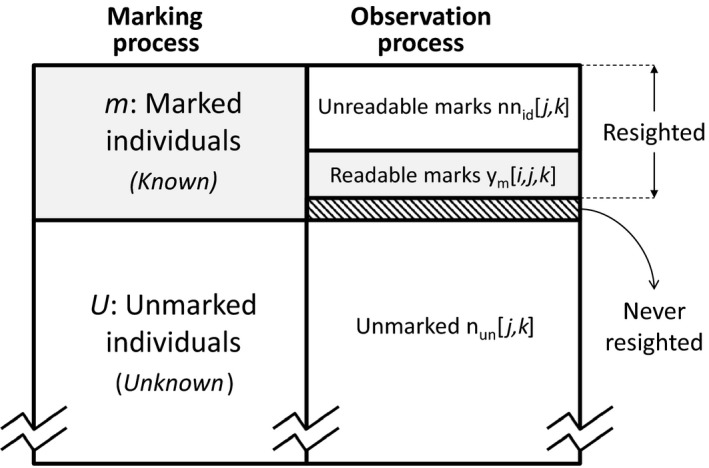
Scheme of spatial mark–resight model and observation process addressed with Generalized SMR with Identification (Gen‐SMR‐ID)

The model is based on the use of the following data from the resighting process: (a) the data on marked and identified individuals; (b) the latent information contained in the counts of marked but unidentified animals; and (c) the latent information about counts from unmarked individuals (marking‐resighting processes and information used in the model are shown in Figure 2).

We have two groups of data, *m* (known) marked and *U* (unknown) unmarked individuals. Population estimate is *N = m + U*.

The model for complete histories from all marked individuals is *y* ^full^ ~Poisson(*λ_ij_*) with:

$$\lambda_{\mathrm{ij}}=\lambda_{0.\text{resight}}\cdot e^{\left(-\frac{d_{\mathrm{ij}}^{2}}{2\sigma^{2}}\right)}.$$

where λ*_ij_* is the encounter rate, *λ*_0.resight_ is the baseline detection (here, resighting) rate, and $d_{\mathrm{ij}}$ the Euclidean distance between activity centers and resighting devices. The core of the Gen‐SMR‐ID model is that we observe only a subset of encounter histories (*y^m^*) from the total encounter histories on marked individuals *y* ^full^: *y^m^* ~Binomial (*y* ^full^,*δ*). The parameter *δ* is the probability of correctly identifying a marked individual that has been detected. The encounter histories of the detected but unidentified marked individuals are a latent variable: *y* ^mu^ = *y* ^full^−*y* ^m^. The information on this latent variable comes from the counts nnid_*jk*_ at each camera trap and each occasion of *m* marked and unrecognizable individuals. Under the binomial observation model, an individual could be in both groups—recognizable and not—on the same sampling occasion, but we can always separate individuals as marked or not marked. The observed data nnid*_jk_* are a sum of latent variables:

$${\text{nnid}}_{\mathrm{jk}}^{\mathrm{un}}=\sum_{i=1}^{m}y_{\mathrm{ijk}}^{\mathrm{mu}}.$$

We used the *dsum* (or the newer *sum*) distribution from JAGS (Plummer, 2003) to deal with latent encounter histories from marked, unidentifiable individuals.

For the unmarked portion of the population, we use the marginal model for the count data, rather than the conditional model based on the latent encounter histories (Royle et al., 2014). We assume the latent encounter histories are Poisson random variables, and we model the observed counts of unmarked individuals as Poisson random variables with an expected value given by the sum (over individuals) of expected encounters:

$$n_{\mathrm{jk}}^{\mathrm{un}}=\sum_{i=1}^{N}y_{\mathrm{ijk}}$$

where *y_ijk_* is another latent variable of those unmarked individuals. Count data are modeled using a Poisson distribution:

$$n_{\mathrm{jk}}^{\mathrm{un}}∼\text{Poisson}\;\left(\Lambda_{j}\right)$$

where:

$$\Lambda_{j}=\lambda_{0.\text{resight}}\sum_{i=1}^{N}e^{\left(-\frac{d_{\mathrm{ij}}^{2}}{2\sigma^{2}}\right)}$$

$\Lambda_{j}$ does not depend on *k*; hence, we can aggregate the *K* occasions of counts:

$$n_{j.}^{\mathrm{un}}∼\text{Poisson}\left(\Lambda_{j}\cdot K\right)$$

This formulation requires information about *λ*_0.resight_ and *σ*, which can be obtained from marked individuals.

#### 2.1.4 Telemetry

This model is based on the use of telemetry data to make *σ* always (and indirectly *λ*_0.resight_) identifiable even in the absence of recognizable individuals. We considered that the home range of each animal came from a bivariate normal movement around its activity center, with a variance *σ* ^2^ (Sollmann, Gardner et al., 2013). According to this, telemetry data from tagged individuals were included in the model to allow inferences about the posterior distribution of *σ*.

#### 2.1.5 State space

The state space was defined as the spatial region *S* including the population of interest large enough to ensure that the encounter rate for an individual whose activity center is located outside the boundary of the region was negligible. We used a minimum of 2.5*σ‐wide buffer around the minimum rectangle envelope defined by the live traps and the camera‐traps. The *σ* value used to build this buffer was calculated in a first run of the model.

### 2.2 Statistical inference

We used Bayesian inference and data augmentation (Royle, Dorazio, & Link, 2007) to deal with the fact that *N* is unknown (Royle et al., 2014). The size (*M*) of the augmented population must be much larger than *N* to not affect the posterior distribution of *N*. For each of the *M* individuals, the latent variable *z* indicates whether the individual was part of the population (1) or not (0). Specifically, *z*_i_∼Bernoulli(φ) implies the relationship *N*∼Binomial(*M*, φ), which indicates whether the individuals from augmented data *M* belong to the population *N*. Population abundance is estimated as the sum of the auxiliary parameter for data augmentation *z*: $N=\sum_{i=1}^{i=M}z_{i}$. Realized population, density is a derived parameter computed from activity center locations in the area of the state space.

### 2.3 Simulations

To assess the relationship between the number of marked individuals, identification rate (δ)—of the probability that a marked individuals is identified—and model accuracy, we ran simulations using a modified version of the original script from Whittington et al. (2017). We simulated a population size of *N = *50 uniformly distributed individuals, with a movement parameter *σ* = 0.05 units, and a grid of 25 live traps (for the marking process) with a distance of 0.15 units. We also simulated 100 camera‐traps (for the resighting process) with a distance of 0.066 units. This trap spacing for the possibility of detecting individuals in more than one camera‐trap. It also induces spatial autocorrelation in the counts of unidentified individuals. In the simulation, every marked individual was GPS‐tagged to use location data in the model to allow or improve σ estimation (Sollmann, Gardner, & Belant, 2012; Sollmann, Gardner et al., 2013).

We used four scenarios: with *m *∈ {5, 10, 15, 20} randomly marked individuals, using a baseline detection (capture) rate *λ*_0.mark_ ∈ {0.05, 0.15, 0.25, 0.35} with 5 marking occasions to enable the simulations with those occasions. We simulated four resighting occasions with *λ*_0.resight_ = 0.5 in all cases. In each scenario, we simulated 10 identification rate values, from 0 to 1, using the same dataset to study the variation in parameter estimates with identification rate for each *m* value. We simulated 50 populations for each pair of values (*m*, *δ*). We fitted our Gen‐SMR‐ID model in a Bayesian framework using the freely available software JAGS (Plummer, 2003) and R (R Core Team, 2018) statistical programming environment using 5,000 iterations, 1,000 adaptations, and a 1,000 burn‐in, keeping the complete posterior estimate for *N*, λ_0.resight_ and *σ*. We calculated the mean, median, and mode for each parameter to compare their performance.

Complete details of the R and JAGS code and data simulator for fitting the Gen‐SMR‐ID model are in Supporting Information Appendix S1.

## 3 APPLICATION TO A RED FOX EMPIRICAL DATASET

### 3.1 Methods

We demonstrate the use of the Gen‐SMR‐ID model to deal with incomplete marked individual identification, using a red fox *Vulpes vulpes* (hereafter, fox) empirical dataset. The fox is the most widespread terrestrial carnivore mammal species and is distributed across the entire northern hemisphere (Macdonald & Reynolds, 2004). It is a generalist and opportunistic predator, including a wide range of foods in its diet (Díaz‐Ruiz et al., 2013). It is an abundant generalist mesopredator in the Mediterranean area (Jiménez, Nuñez‐Arjona et al., 2017). Conflicts with human interests are common due to its predation on small game species, livestock, and ground‐nesting birds of conservation concern (Bolton, Tyler, Smith, & Bamford, 2007; Fernandez‐de‐Simon et al., 2015; Greentree, Saunders, Mcleod, & Hone, 2000; Reynolds & Tapper, 1995). Red foxes act as seed dispersers for many fruit species in the Mediterranean region and can potentially influence the community composition of several habitats (Cancio et al., 2017). Therefore, the estimation of red fox abundance is of great interest from ecological, conservation, and management points of view. Red foxes lack clear pelage patterns that would otherwise allow for the identification of individuals (Güthlin, Storch, & Küchenhoff, 2014) (but see Sarmento, Cruz, Eira, & Fonseca, 2009). As a part of an experimental study on fox predation, we deployed two grids of live traps (Collarum^©^) and camera‐traps in two areas of Ciudad Real (Central Spain). La Nava (Almagro municipality) study area was covered by a mixture of Mediterranean scrubland, sparse patches of holm oak *Quercus ilex* subsp. *rotundifolia* and cereal fields, with a high density of European rabbit *Oryctolagus cuniculus*. The study area was 14.66 km^2^, defined by the envelope around detector devices. The other study area, Los Pilones (Abenojar and Saceruela municipalities) was covered with Mediterranean dense scrub (i.e., gum rockrose *Cistus ladanifer*) with a scanty rabbit population. The study area around detector devices was 23.26 km^2^.

Live traps were baited with a commercial Collarum attractant with pork and chicken bait plus lynx urine (collected from captive lynx breeding facilities) and were checked every morning. Captured foxes were anesthetized with medetomidine hydrochloride (Medetor^®^, Virbac, Spain) and ketamine hydrochloride (Imalgene 1000^®^, Merial, Spain). Foxes were tagged with GPS radio‐collars (MiniTrack^®^, Lotek, Ontario, Canada) and two models of numbered ear tags, one sheep commercial ear tag (3.4 × 3.2 cm) (Cromasa^®^, Berriozar, Navarra, Spain) and an ear tag specifically designed for red fox (6.8 × 3.2 cm) (Maquia Serveis Ambientals^®^, Alcoi, Alicante Spain).

In La Nava, we used 22 live traps over 51 days (9 December 2015 to 28 January 2016). In Los Pilones, we used 50 live traps over 35 days (28 January 2016 to 2 February 2016). Live trap locations were selected to maximize capture of foxes (e.g., in the trails usually used by this species).

The camera‐traps used were Spartan SR1‐BK^®^ HCO Outdoor Products, Norcross, Georgia, USA and Reconyx HC500 Hyperfire Semi‐Covert IR^®^, Holmen, Wisconsin, USA. In La Nava, we used 49 camera‐traps over 89 days (27 January 2016 to 24 April 2016). Mean distance to the nearest neighboring camera‐traps was 133.8 m. In Los Pilones, we used 33 camera‐traps over 63 days (12 March 2016 to 13 April 2016) with a mean distance to the nearest neighboring trap of 387.8 m (Supporting Information Appendix S2). Those distances are below the σ value reported for fox (Jiménez, Nuñez‐Arjona et al., 2017) allowing spatial correlation between captures. Camera‐traps were placed at sites suitable to detect animals and were baited with red‐legged partridge (*Alectoris rufa*) eggs. Cameras were visited approximately every 7 days to replace the bait, perform camera maintenance, and download data. We programmed Spartan cameras to record 10 s videos with a minimum time delay (0 s) between consecutive records. The Reconyx cameras, which do not allow video mode, were programmed in RapidFire^TM^ mode (two frames per second) to maximize the number of photos taken per captured individual. Consecutive images of foxes within 30 min intervals were considered as the same event, whereas those separated by longer intervals were considered as independent events. If there were more than one individual in the same picture, we considered one event for each individual (Jiménez, Nuñez‐Arjona et al., 2017). Pictures for which we could not distinguish whether an individual was marked were discarded.

GPS radio‐collars were scheduled to take one position per hour during the night and one location every 2 hr during the day. GPS collars weighed 163 g plus 42 g for the optional drop‐off release mechanism. The drop‐off system was used only on heavier foxes so that the total device (collar + drop‐off) was always lighter than 5% body weight (range: 2.6%–4.2%). The drop‐off system was scheduled to activate 26 weeks after tagging. Foxes without drop‐off systems were recaptured after 26 weeks for removal of the GPS collars. GPS data were downloaded regularly with a VHF remote receptor or after recapture. After collar recovery, foxes were released in the same location in which they were captured.

### 3.2 Model specifications

We used a Uniform (0, 1) prior for baseline detection rate in the marking process (λ_0.mark_) under a binomial observation model, and a Uniform (0, 2) prior for the baseline detection rate for resighting (λ_0.resight_) modeled with a Poisson. The prior probability of the parameter for data augmentation (*φ*) is a Uniform (0, 1), which is equivalent to assuming a uniform prior between 0‐*M*.

To define *S* (the state space), in each case, we used an envelope from the detector devices polygon, plus an additional buffer of 2.5*σ. Buffer sizes were estimated using sigma values in a first running of both codes.

The number of marked individuals (*m*) used was the total captured in the marking process for which GPS devices indicated that they were alive during the resighting process. Capture histories for marked but undetected individuals were included as all zeros. One animal died during the resighting period. To deal with this loss, avoiding bias in the detection process by introducing individuals that could not be detected, we used a binary variable of life [*i*, *k*] (individual × occasion) with one for live occasions and zero when we had evidence that the animal was dead.

After the first trial, we settled on *M* = 150 for data augmentation. In the model for La Nava, we ran three parallel chains for 85,000 iterations, 5,000 adaptions and discarded the first 5,000 as burn‐in. In the model for Los Pilones, we ran three parallel chains for 52,500 iterations, 1,500 adaptions and discarded the first 2,500 as burn‐in. Gelman–Rubin diagnostic statistics and visual inspections were used to assess convergence. The models were fitted using a script written in JAGS and R (Supporting Information Appendix S1).

We compared the outputs from La Nava and Los Pilones with the same dataset but simulating all marked individuals as unidentified, in order to compare the accuracy of estimates under different identification rates.

We also used a distance sampling approach with a fully independent dataset to validate the inferences from our new Gen‐SMR‐ID model. Distance sampling was successfully applied in La Nava, where visual detectability is very high due to nearly no scrub cover and high fox density. However, line transects in Los Pilones prevented the application of distance sampling methodology: No foxes were seen in a test of two replicates of the same transect of 22,080 m due to low visibility because of vegetation thickness. We used three transects in La Nava study area and five temporal replicates on different days (from October to November 2015, before live trapping). Each detected animal was recorded, and its distance and azimuth from the sampling point was measured using a laser range finder and magnetic compass. Perpendicular distances were calculated, and distance break classes were set to 50 m. The maximum observation distance was 366 m. We used the package *unmarked* (Fiske & Chandler, 2011) in R (R Core Team, 2018). We selected best‐approximating models of abundance and detection probability, using a model with no covariates for a null estimate considering three detection functions (half‐normal, hazard rate, and exponential) and two abundance distributions (Poisson and negative binomial) for each group. We ranked null models using the Akaike information criterion (AIC; Burnham & Anderson, 2002) considering the model with the lowest AIC score to be the best‐fitting key function and distribution. We used the parametric bootstrap approach in *unmarked* to obtain *p*‐values from sums of squares (SSE), Chi‐square test, and Freeman‐Tukey fit statistics to quantify the fit of a model to a dataset.

### 3.3 Results

In our simulations, the root mean square error (RMSE) for population size estimation for *m* = 5 ranged from 8.4 (*δ*: 0) to 8.6 (*δ*: 1), and for *m* = 20, from 4.3 (*δ*: 0) to 4.24(*δ*: 1). The RMSE indicates that the best estimator for the population size for *m* = 5 is the mode. The RMSE for mean is lower for a higher number of marked individuals (Table 1). For other parameters, the behavior of *σ* is notable (Supporting Information Appendix S3); although the bias in the model is very small for *m* = 5 (see axes), it is unbiased if *δ* > 0.2. For *m* > 5, the σ estimate is unbiased in all cases.

**Table 1:** Posterior mean, median, mode, and coverage rates for the 95% highest posterior density (HPD) interval for simulations from a population of *N* = 50 individuals in which *m* ∈ {5, 10, 15, 20} were marked. δ: identification rate. Fifty simulations of each case were conducted

Ind. Marked	δ	Mean	RMSE	Median	RMSE	Mode	RMSE	Coverage
m = 5	0.00	50.06	8.41	49.22	8.25	47.54	8.46	0.98
	0.50	50.62	8.46	49.80	8.32	48.29	8.02	0.98
	1.00	50.95	8.63	50.03	8.37	48.38	8.16	0.98
m = 10	0.00	49.10	7.37	48.60	7.35	47.63	7.62	1.00
	0.50	49.42	7.29	48.86	7.32	47.98	7.49	1.00
	1.00	49.39	7.28	48.89	7.30	48.08	7.32	1.00
m = 15	0.00	48.14	5.69	47.70	5.83	46.87	6.23	0.98
	0.50	48.34	5.60	47.92	5.71	47.22	6.12	0.98
	1.00	48.32	5.57	47.86	5.78	47.12	6.14	0.98
m = 20	0.00	49.12	4.30	48.70	4.42	48.70	4.46	1.00
	0.50	49.19	4.25	48.82	4.25	48.82	4.67	1.00
	1.00	49.19	4.24	48.79	4.33	48.09	4.57	1.00

In our case studies, we marked eight individuals in La Nava over 457 trap‐days (1.75 captures/100 trap‐days). In the resighting process (1,325 camera‐days), we had 119 capture events with camera‐traps (8.98 captures/100 camera‐days) with 32 events of marked individuals, but only two individuals resighted and identified in six events, and 87 events of unmarked individuals. Were obtained 4,229 GPS locations from six individuals.

We marked five individuals in Los Pilones over 545 trap‐days: 1.1 captures/100 trap‐days. In the resighting process (862 camera‐days), we had 124 capture events with camera‐traps (14.39 captures/100 camera‐days) with 111 events of marked individuals; 102 events corresponded to identified individuals. We had 83 events with readable marks and 19 events with unreadable marks, but with foxes that were identifiable by individual traits. We additionally identified the individuals in those 19 events using a complete coincidence among 3 researchers, as we aimed to compare two extreme cases of identification rate (La Nava‐Los Pilones). We also had nine events of two marked and unidentified individuals, and 13 events of unmarked individuals. We resighted all five marked individuals. We gathered 1,547 GPS locations from three individuals.

Gelman–Rubin statistics were <1.1 for all parameters and visual inspections of the trace plots indicated that the Markov chains successfully converged (Supporting Information Appendix S4).

Results are shown in Table 2. Density estimates of mean = 1.60 (1.04–2.31) and 0.28 (0.18–0.27) individuals/km^2^ were obtained in La Nava and Los Pilones, respectively. The coefficient of variation (CV) for both estimates was 0.20. With no marked individuals identified (and using GPS data), we would have obtained a density of mean = 1.64 (1.02–2.44) and 0.27 (0.18–0.39) individuals/km^2^ in La Nava and Los Pilones, with a CV($\hat{N}$) = 0.23 and 0.19, respectively (Supporting Information Appendix S5).

**Table 2:** Posterior mean, standard deviation, and 95% HPD interval coverage of model parameters from the generalized spatial mark–resight with incomplete identification model (Gen‐SMR‐ID) from red fox (*Vulpes vulpes*) case studies in La Nava and Los Pilones (Ciudad Real, Central Spain)

	Mean	*SD*	2.50%	50%	97.50%
La Nava					
lam0.mark (*λ*_0.mark_)	0.02	0.01	0.01	0.02	0.04
lam0.resight (*λ*_0.resight_)	0.07	0.01	0.04	0.06	0.09
sigma (*σ*)	0.43	0.00	0.42	0.43	0.44
psi (ψ)	0.39	0.09	0.24	0.38	0.57
$\hat{N}$	58.08	11.72	38.00	57.00	84.00
$\hat{D}$	1.60	0.32	1.04	1.57	2.31
*δ*	0.21	0.07	0.09	0.20	0.36
Deviance	10,304.66	12.36	10,281.82	10,304.20	10,330.17
Los Pilones					
lam0.mark (*λ*_0.mark_)	0.03	0.01	0.01	0.03	0.06
lam0.resight (*λ*_0.resight_)	0.46	0.06	0.36	0.46	0.58
sigma (*σ*)	0.53	0.01	0.52	0.53	0.55
psi (ψ)	0.12	0.03	0.06	0.11	0.19
$\hat{N}$	16.86	3.39	11.00	16.00	24.00
$\hat{D}$	0.28	0.06	0.18	0.27	0.40
*δ*	0.91	0.03	0.85	0.91	0.96
Deviance	5,020.25	10.05	5,001.90	5,019.77	5,041.42

Baseline detection rate for capture (*λ*_0.mark_), baseline detection rate for resighting (*λ*_0.resight_), parameter of movement (*σ*), data augmentation parameter (ψ), population size estimate in the state space ($\hat{N}$), density estimate ($\hat{D}$), identification probability (*δ*) and deviance.

### 3.4 Test of results using distance sampling

Our five temporal replicates of transects, with no evidence of temporary emigration between counts, were simply replicated counts, and consequently, we stacked the data replication for analysis (Flockhart, Norris, & Coe, 2016) to reduce variation in estimating the population size. We estimated a density of 1.3 (0.57–2.03) individuals/km^2^ in La Nava using distance sampling methodology (Supporting Information Appendix S6).

The bootstrap *p*‐values for the best‐fitting model based on the SSE, Freeman‐Tukey, and Chi‐square statistics were 0.42, 0.34, and 0.52, respectively, suggesting that our model adequately fits the data. The value of *ĉ* (ratio of observed/expected) was 1.04.

## 4 DISCUSSION

Spatial mark–resight models are a useful approach to estimate population densities of species lacking natural markings that are detected using camera‐traps. SMR is based on marking a portion of the population and later collecting data from both marked and unmarked individuals.

We developed an extension of the generalized spatial mark–resight model (Gen‐SMR‐ID) to deal with a very common problem: the difficulty of reading all the marks and recognizing individuals using camera‐traps. Royle et al. (2014) described a solution for this problem in SMR, that was to use a correction factor *c* considering that the number of identified events is a binomial distribution of the total events with probability *c*: $\sum y_{c}∼\text{Binomial}\left(\sum y_{m},c\right)$, where *y_c_* are the correctly identified individuals and *y_m_* all marked individuals (identified or not). However, this approach does not make full use of the spatial information of the marked and unidentified records.

Gen‐SMR‐ID allows inferences about population size, even without individual identification of the marked animals. However, model performance will depend on the number of marked individuals and the availability of telemetry data. Our simulation study indicated that performance was satisfactory when at least 10% of the population was marked and outfitted with telemetry devices, even when the individual identification rate was low. This result suggests that the spatially autocorrelated counts of marked but unidentified individuals are informative about resighting parameters, and this information results in improved estimates of abundance and density. We need further studies about the identifiability on this model without telemetry data.

Our simulation study demonstrated that posterior precision increases, as expected, with the number of marked individuals. Coverage rates for the 95% highest posterior density (HPD) intervals were close to nominal for all values of *m* studied (Table 1).

**Figure 3:**
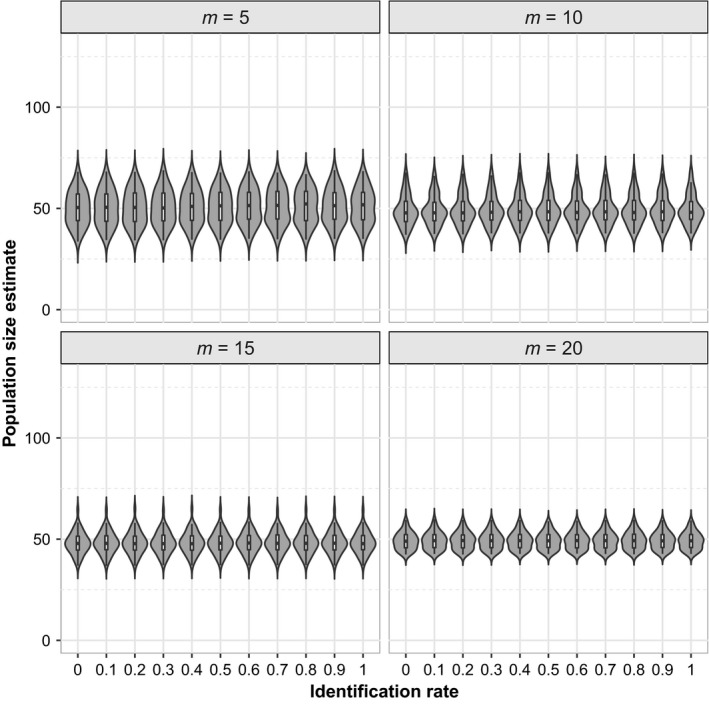
Posterior means violin plot of abundance (*N* = 50) estimates using generalized spatial mark–resight with incomplete identification model (Gen‐SMR‐ID) in JAGS from different number of marked individuals, *m *∈ {5, 10, 15, 20}, plotted against the identification rate (δ)

The posterior mean, median, and mode exhibited low bias when used as a point estimate of *N*. Surprisingly, we found that estimates of population size were unbiased and precise even when the identification rate (*δ*) was zero (Table 2, Figure 3). The use of telemetry data makes *σ* (and *λ*_0_) identifiable.

In our study case, the model was used to estimate fox population in high‐ and low‐density scenarios. An additional comparison of the estimate was carried out in the high‐density area using distance sampling. Using the Gen‐SMR‐ID model, the population size estimates are almost the same with or without individual identification (Table 2 and Supporting Information Appendix S5), for both the high‐density case, with a posterior probability of identification  = 0.21 (0.07–0.36) (Table 1), and for the low‐density case, with a posterior probability of identification  = 0.91 (0.85–0.96) (Table 2). The coefficient of variation obtained (ca 0.2) is consistent with the results of the simulations, considering the rate of animals that are marked (0.14 and 0.29, respectively).

Agreement with the results of the distance sampling was strong (Table 2 and Supporting Information Appendix S6) although the Gen‐SMR‐ID model showed lower standard deviation. Attention should be paid to the fact that the estimation of fox abundance with distance sampling is seldom feasible in Mediterranean habitats due to low visibility. The very open habitat of La Nava, and its high fox density, allowed a sufficient number of detections for the application of distance sampling. Los Pilones presented the opposite, and more common, scenario. Overall, generating precise and accurate abundance estimates of fox populations continues to be challenging, and thus SMR presents a useful approach.

The negative relationship between the population sizes estimated in the two study areas (1.60 and 0.28 foxes/km^2^ in La Nava and Los Pilones, respectively), and the capture index (8.98 and 14.39 events/100 camera‐days), is notable. The higher capture index in Los Pilones may be related to the smaller amount of available food, which would exacerbate search behavior, and thus the number of camera‐trapping events. These results support previous studies calling attention to the risk of using raw capture indices from camera‐trap data as a measure of relative abundance (Sollmann, Mohamed, Samejima, & Wilting, 2013). They highlight the need for reliable methods for density estimation of cryptic species, such as the Gen‐SMR‐ID model proposed here. Our study case is a clear example of this risk; considering only raw capture indices, we would have concluded that a higher density population occurs in Los Pilones than in La Nava, when the opposite is true according to our Gen‐SMR‐ID models. Even so, studies continue using relative abundance indices, mostly because sound statistical methods for population estimation are not available or cannot be applied for the species of concern. Our proposed Gen‐SMR‐ID approach provides a valuable tool for researchers to address this gap and will hopefully help reduce the inappropriate use of raw abundance indices in camera‐trapping studies with individually unrecognizable species.

The results for fox density are consistent with those of other authors in Mediterranean areas. Sarmento et al. (2009) estimated densities of 0.61 (0.54–0.69) individuals/km^2^ in Serra de Malcata (Portugal) using nonspatial capture–recapture methods and identifying all individuals by natural marks. Jiménez, Nuñez‐Arjona et al. (2017) estimated 0.41 (0.21–0.72) individuals/km^2^ using SMR in southern Spain.

This model can be extended to other formulations of SMR, such as temporal or behavioral variation in baseline detection rate, and spatial variation in density, or using categorical covariates (Augustine et al., 2018). We can recognize in camera‐trap pictures some categorical identities like “male, subadult” or “female, adult” allowing increased precision when data are sparse and/or there are few marked individuals. It also can be used with naturally marked populations, using as a marked population the recognizable individuals in a previous (“marking”) period but we will need spatial recaptures or additional telemetry data to improve the estimates of abundance and density.

## CONFLICT OF INTEREST

None declared.

## AUTHOR CONTRIBUTIONS

JJ and RC conceived the ideas and designed methodology; PF, JT, RM, and ED collected the data; JJ analyzed the data; JJ and PF led the writing of the manuscript. All authors contributed critically to the drafts and gave final approval for publication.

## Data Availability

All data and code generated for analysis will be available in the PANGAEA® repository following manuscript acceptance (https://doi.pangaea.de/10.1594/PANGAEA.898560).

## References

[ece35077-bib-0001] Augustine, B. C. , Royle, J. A. , Stewart, F. E. C. , Fisher, J. T. , & Kelly, M. J. (2018). Spatial mark‐resight for categorically marked populations with an application to genetic capture‐recapture. BioRxiv, 299982 [Preprint]. 10.1101/299982

[ece35077-bib-0002] Bolton, M. , Tyler, G. , Smith, K. , & Bamford, R. (2007). The impact of predator control on lapwing *Vanellus vanellus* breeding success on wet grassland nature reserves. Journal of Applied Ecology, 44(3), 534–544. 10.1111/j.1365-2664.2007.01288.x

[ece35077-bib-0003] Borchers, D. L. , & Efford, M. G. (2008). Spatially explicit maximum likelihood methods for capture‐recapture studies. Biometrics, 64, 377–385. 10.1111/j.1541-0420.2007.00927.x 17970815

[ece35077-bib-0004] Burnham, K. P. , & Anderson, D. R. (2002). Model selection and multimodel inference: a practical information‐theoretic approach, 2nd ed Ecological Modelling (Vol. 172). Berlin, Germany: Springer Verlag 10.1016/j.ecolmodel.2003.11.004

[ece35077-bib-0005] Cancio, I. , González‐Robles, A. , Bastida, J. M. , Isla, J. , Manzaneda, A. J. , Salido, T. , & Rey, P. J. (2017). Landscape degradation affects red fox (*Vulpes vulpes*) diet and its ecosystem services in the threatened *Ziziphus lotus* scrubland habitats of semiarid Spain. Journal of Arid Environments, 145, 24–34. 10.1016/j.jaridenv.2017.05.004

[ece35077-bib-0006] Chandler, R. B. , & Royle, J. A. (2013). Spatially‐explicit models for inference about density in unmarked populations. The Annals of Applied Statistics, 7(2), 936–954. 10.1214/12-AOAS610

[ece35077-bib-0007] Díaz-Ruiz, F. , Delibes-Mateos, M. , García-Moreno, J. L. , María López-Martín, J. , Ferreira, C. , & Ferreras, P. (2013). Biogeographical patterns in the diet of an opportunistic predator: The red fox *Vulpes vulpes* in the Iberian Peninsula. Mammal Review, 43, 59–70. 10.1111/j.1365-2907.2011.00206.x

[ece35077-bib-0008] Efford, M. G. , Dawson, D. K. , & Robbins, C. S. (2004). DENSITY: Software for analysing capture‐recapture data from passive detector arrays. Animal Biodiversity and Conservation, 27(1), 217–228.

[ece35077-bib-0009] Efford, M. G. , & Hunter, C. M. (2018). Spatial capture–mark–resight estimation of animal population density. Biometrics, 74(2), 411–420. 10.1111/biom.12766 28834536

[ece35077-bib-0010] Fernandez‐de‐Simon, J. , Díaz‐Ruiz, F. , Rodríguez‐de la Cruz, M. , Delibes‐Mateos, M. , Villafuerte, R. , & Ferreras, P. (2015). Can widespread generalist predators affect keystone prey? A case study with red foxes and European rabbits in their native range. Population Ecology, 57(4), 591–599. 10.1007/s10144-015-0510-5

[ece35077-bib-0011] Fiske, I. J. , & Chandler, R. B. (2011). unmarked: An R package for fitting hierarchical models of wildlife occurrence and abundance. Journal of Statistical Software, 43(10), 4739–23. 10.1002/wics.10

[ece35077-bib-0012] Flockhart, D. T. T. , Norris, D. R. , & Coe, J. B. (2016). Predicting free‐roaming cat population densities in urban areas. Animal Conservation, 19(5), 472–483. 10.1111/acv.12264

[ece35077-bib-0013] Green, R. F. , Otis, D. L. , Burnham, K. P. , White, G. C. , & Anderson, D. R. (1978). Statistical inference from capture data on closed animal populations. Wildlife Monographs, 62(62), 3–135. 10.2307/2287873

[ece35077-bib-0014] Greentree, C. , Saunders, G. R. , Mcleod, L. , & Hone, J. (2000). Lamb predation and control of foxes in south‐eastern Australia. Journal of Applied Ecology, 37, 935–943. 10.1046/j.1365-2664.2000.00530.x

[ece35077-bib-0015] Güthlin, D. , Storch, I. , & Küchenhoff, H. (2014). Is it possible to individually identify red foxes from photographs? Wildlife Society Bulletin, 38(1), 205–210. 10.1002/wsb.377

[ece35077-bib-0016] Jiménez, J. , Higuero, R. , Charre‐Medellín, J. F. , & Acevedo, P. (2017). Spatial mark‐resight models to estimate feral pig population density. Hystrix, the Italian Journal of Mammalogy Online, 28(2), 208–213. 10.4404/hystrix-28.2-12141.

[ece35077-bib-0017] Jiménez, J. , Nuñez‐Arjona, J. C. , Rueda, C. , González, L. M. , García‐Domínguez, F. , Muñoz‐Igualada, J. , & López‐Bao, J. V. (2017). Estimating carnivore community structures. Scientific Reports, 7(1), 4739–4748. 10.1038/srep41036 28120871 PMC5264395

[ece35077-bib-0018] Karanth, K. U. , & Nichols, J. D. (1998). Estimation of tiger densities in India using photographic captures and recaptures. Ecology, 79, 2852–2862. 10.2307/176521

[ece35077-bib-0019] Kéry, M. , Gardner, B. , Stoeckle, T. , Weber, D. , & Royle, J. A. (2011). Use of spatial capture‐recapture modeling and DNA data to estimate densities of elusive animals. Conservation Biology, 25(2), 356–364. 10.1111/j.1523-1739.2010.01616.x 21166714

[ece35077-bib-0020] Macdonald, D. W. , & Reynolds, J. C. (2004). Red fox *Vulpes vulpes* Linnaeus, 1758 In Sillero-Zubiri C., Hoffmann M., & Macdonald D. W. (Eds.), Canids: Foxes, wolves, jackals and dogs. Status survey and conservation action plan (pp. 129–136). Gland, Switzerland: IUCN/SSC Canid Specialist Group.

[ece35077-bib-0021] McClintock, B. T. , Hill, J. M. , Fritz, L. , Chumbley, K. , Luxa, K. , & Diefenbach, D. R. (2014). Mark‐resight abundance estimation under incomplete identification of marked individuals. Methods in Ecology and Evolution, 5(12), 1294–1304. 10.1111/2041-210X.12140

[ece35077-bib-0022] Parmenter, R. R. , Yates, T. L. , Anderson, D. R. , Burnham, K. P. , Dunnun, J. L. , Franklin, A. B. , White, G. C. (2003). Small‐mammal density estimation: A field comparison of grid‐based vs. web‐based density estimators. Ecological Monographs, 73(1), 4739–26. 10.1890/0012-9615(2003)073[0001:SMDEAF]2.0.CO;2

[ece35077-bib-0023] Plummer, M. (2003). JAGS: A program for analysis of Bayesian graphical models using Gibbs sampling. 3rd International Workshop on Distributed Statistical Computing (DSC 2003). Vienna, Austria.

[ece35077-bib-0024] R Core Team (2018). R: A language and environment for statistical computing. Vienna, Austria: R Foundation for Statistical Computing.

[ece35077-bib-0025] Reynolds, J. C. , & Tapper, S. C. (1995). The ecology of the red fox *Vulpes* *vulpes* in relation to small game in rural southern England. Wildlife Biology, 1(1), 105–119. 10.2981/wlb.1995.0016

[ece35077-bib-0026] Royle, J. A. , Chandler, R. B. , Sollmann, R. , & Gardner, B. (2014). Spatial capture‐recapture. Waltham, MA: Elsevier, Academic Press 10.1016/B978-0-12-405939-900026-8

[ece35077-bib-0027] Royle, J. A. , Dorazio, R. M. , & Link, W. A. (2007). Analysis of multinomial models with unknown index using data augmentation. Journal of Computational and Graphical Statistics, 16(1), 67–85. 10.1198/106186007X181425

[ece35077-bib-0028] Royle, J. A. , & Young, K. (2008). A hierarchical model for spatial capture‐recapture data. Ecology, 89(8), 2281–2289. 10.1890/07-0601.1 18724738

[ece35077-bib-0029] Sarmento, P. B. , Cruz, J. P. , Eira, C. I. , & Fonseca, C. (2009). Evaluation of camera trapping for estimating red fox abundance. Journal of Wildlife Management, 73(7), 1207–1212. 10.2193/2008-288

[ece35077-bib-0030] Silvy N. J. (Ed.) (2012). The wildlife techniques manual, 7th ed Baltimore, MD: Johns Hopkins University Press.

[ece35077-bib-0031] Soisalo, M. K. , & Cavalcanti, S. M. C. C. (2006). Estimating the density of a jaguar population in the Brazilian Pantanal using camera‐traps and capture‐recapture sampling in combination with GPS radio‐telemetry. Biological Conservation, 129(4), 487–496. 10.1016/j.biocon.2005.11.023

[ece35077-bib-0032] Sollmann, R. , Gardner, B. , & Belant, J. L. (2012). How does spatial study design influence density estimates from spatial capture‐recapture models? PLoS ONE, 7, e34575 10.1371/journal.pone.0034575 22539949 PMC3335117

[ece35077-bib-0033] Sollmann, R. , Gardner, B. , Parsons, A. W. , Stocking, J. J. , McClintock, B. T. , Simons, T. R. , … O'Connell, A. F. (2013). A spatial mark‐resight model augmented with telemetry data. Ecology, 94(3), 553–559. 10.1890/12-1256.1 23687880

[ece35077-bib-0034] Sollmann, R. , Mohamed, A. , Samejima, H. , & Wilting, A. (2013). Risky business or simple solution – Relative abundance indices from camera‐trapping. Biological Conservation, 159, 405–412. 10.1016/j.biocon.2012.12.025

[ece35077-bib-0035] Whittington, J. , Hebblewhite, M. , & Chandler, R. B. (2017). Generalized spatial mark‐resight models with an application to grizzly bears. Journal of Applied Ecology, 55(1), 157–168. 10.1111/1365-2664.12954

