# Corrigendum

**DOI:** 10.1002/ece3.5647

**Published:** 2019-10-11

In “Generalized spatial mark–resight models with incomplete identification: An application to red fox density estimates” which was published in Volume 9, Issue 8, April 2019, the authors would like to draw the attention of the reader to the following:

In the code, we used the JAGS function dsum(). We have found this function did not correctly sample from the posterior distribution of y.full. We reran the same GEN‐SMR‐ID code using the JAGS function sum() instead, as suggested as an alternative in the article, and it worked properly. Even though dsum() did not work correctly, results using sum() and dsum() were almost the same because we used initial values that were close to the actual values of y.full (Table 1). New outputs from La Nava and Los Pilones are in Table 2.

**Table 1:** Posterior mean, median, mode, and coverage rates for the 95% highest posterior density (HPD) interval for simulations from a population of *N* = 50 individuals in which *m* ∈ {5, 10, 15, 20} were marked. *δ*: identification rate. Fifty simulations of each case were conducted (using sum() function)

Ind. marked	δ	Mean	RMSE	Median	RMSE	Mode	RMSE	Coverage
*m* = 5	0.00	51.04	8.63	50.20	8.40	48.61	8.35	0.98
	0.50	51.02	8.66	50.12	8.45	48.49	8.59	0.98
	1.00	50.86	8.52	50.05	8.22	48.40	8.14	0.98
*m* = 10	0.00	49.44	7.20	48.90	7.13	47.62	7.31	1.00
	0.50	49.51	7.28	48.97	7.30	48.09	7.49	1.00
	1.00	49.38	7.28	48.92	7.33	47.96	7.53	1.00
*m* = 15	0.00	48.18	5.62	47.72	5.68	46.99	6.00	0.98
	0.50	48.32	5.60	47.86	5.76	47.00	6.01	0.98
	1.00	48.35	5.58	47.94	5.70	47.13	6.03	0.98
*m* = 20	0.00	49.32	4.29	48.94	4.32	48.20	4.59	1.00
	0.50	49.27	4.25	48.88	4.37	48.12	4.74	1.00
	1.00	49.24	4.29	48.87	4.31	48.12	4.48	1.00

**Table 2:** Posterior mean, standard deviation, and 95% HPD interval coverage of model parameters from the generalized spatial mark–resight with incomplete identification model (using sum() function) from red fox (*Vulpes vulpes*) case studies in La Nava and Los Pilones (Ciudad Real, Central Spain). lam0.mark (λ_0.mark_) and lam0.resight (λ_0.resight_): basal detection (marking and resighting), sigma (σ), and psi (ψ): movement and data augmentation parameters; *N*: population size, *D*: density and *δ*: identification rate

	Mean	SD	2.50%	50%	97.50%
La Nava					
lam0.mark (λ_0.mark_)	0.03	0.01	0.01	0.02	0.05
lam0.resight (λ_0.resight_)	0.08	0.02	0.05	0.06	0.12
sigma (σ)	0.43	0.00	0.42	0.43	0.43
psi (ψ)	0.35	0.08	0.20	0.34	0.52
*N*	51.50	10.77	33.00	51.00	75.00
*D*	1.41	0.30	0.91	1.40	2.10
*δ*	0.21	0.07	0.09	0.20	0.35
Los Pilones					
lam0.mark (λ_0.mark_)	0.04	0.01	0.01	0.03	0.07
lam0.resight (λ_0.resight_)	0.49	0.06	0.39	0.49	0.61
sigma (σ)	0.53	0.01	0.52	0.53	0.54
psi (ψ)	0.12	0.03	0.06	0.11	0.19
*N*	16.85	3.35	11.00	16.00	24.00
*D*	0.28	0.06	0.18	0.27	0.40
*δ*	0.91	0.03	0.85	0.91	0.96

Because the correction did not greatly change our results, the main conclusions and findings reported in the Discussion still stand. However, we would like to emphasize that users should use sum() instead of dsum().
