# Supplementary material for: Generalized spatial mark–resight models with incomplete identification: An application to red fox density estimates
Source: Ecol Evol. 2019 Mar 22;9(8):4739–48. doi: 10.1002/ece3.5077 (PMC6476752; doi:10.1002/ece3.5077)
Supplement: Supplementary file 1 [file ECE3-9-4739-s001.pdf]

Supporting Information S1: R code to simulate data and analyze them with JAGS

Generalized Spatial Mark-Resight models with incomplete identification: an application to red fox density estimates

José Jiménez[1], Richard Chandler[2], Jorge Tobajas[1], Esther Descalzo[1], Rafael Mateo[1] and Pablo Ferreras[1]

[1] Instituto de Investigación en Recursos Cinegéticos (IREC, CSIC-UCLM-JCCM), Ronda de Toledo 12, 13071 Ciudad Real, Spain.
[2] University of Georgia, Warnell School of Forestry and Natural Resources.

Modified from: Whittington, J., Hebblewhite, M., & Chandler, R. B. (2017). Generalized spatial mark-resight models with an application to grizzly bears. *Journal of Applied Ecology*, (November 2016), 1-12. doi:10.1111/1365-2664.12954

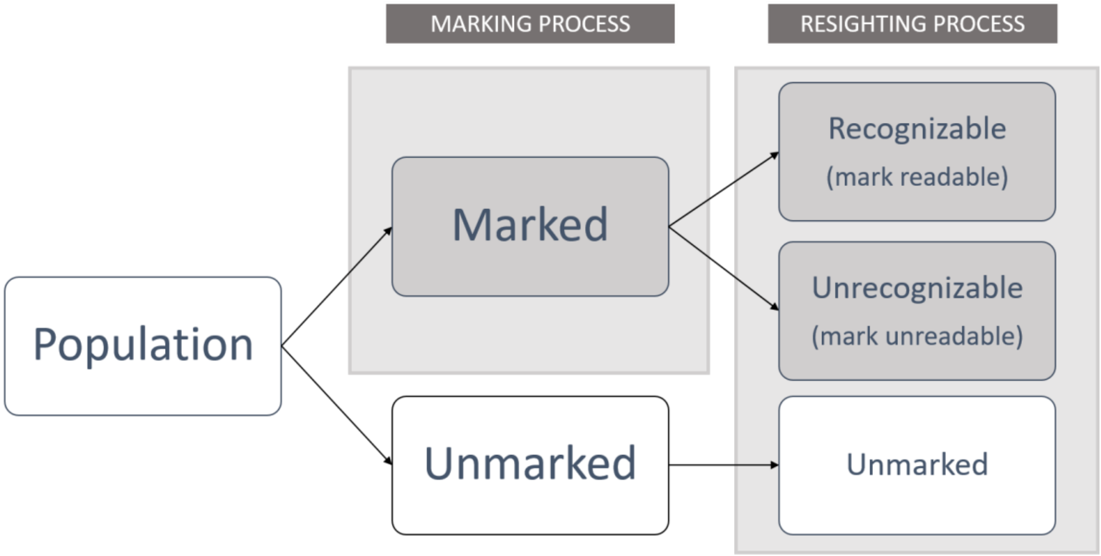

Figure 1. Gen-SMR-ID script scheme

Table of Contents

Define working directory and load some libraries 2

Funtions to use 3

Data simulator 3

BUGS model 6

Data simulation 7

Fixing identification rate 9

Data 9

Inits 10

Parameters to save 10

Run JAGS 11

Define working directory and load libraries

```
setwd('C:/…')
library(stringr)
library(dplyr)
##
## Attaching package: 'dplyr'
## The following objects are masked from 'package:stats':
##
##     filter, lag
## The following objects are masked from 'package:base':
##
##     intersect, setdiff, setequal, union
library(tidyr)
library(coda)
library(raster)
## Loading required package: sp
##
## Attaching package: 'raster'
## The following object is masked from 'package:tidyr':
##
##     extract
## The following object is masked from 'package:dplyr':
##
##     select
library(rgeos)
## rgeos version: 0.3-28, (SVN revision 572)
##  GEOS runtime version: 3.6.1-CAPI-1.10.1 r0
##  Linking to sp version: 1.3-1
##  Polygon checking: TRUE
library(ggplot2)
library(mvtnorm)
library(jagsUI)
## Loading required package: lattice
##
## Attaching package: 'jagsUI'
## The following object is masked from 'package:coda':
##
##     traceplot
```

```
## The following object is masked from 'package:utils':
##
##     View
library(scrbook)
##
## Attaching package: 'scrbook'
## The following object is masked from 'package:raster':
##
##     area
set.seed(1960)
```

Functions to use

```
e2dist <- function (x, y) {  # Function from scrbook package to calculate the
                             # distance between locations in 2 matrices.
  i <- sort(rep(1:nrow(y), nrow(x)))
  dvec <- sqrt((x[, 1] - y[i, 1])^2 + (x[, 2] - y[i, 2])^2)
  matrix(dvec, nrow = nrow(x), ncol = nrow(y), byrow = F)
}

e2dist.2 <- function (x, y) {  # Function from scrbook package to calculate
                               # the squared distance between locations in
                               # 2 matrices.
  i <- sort(rep(1:nrow(y), nrow(x)))
  dvec <- (x[, 1] - y[i, 1])^2 + (x[, 2] - y[i, 2])^2
  matrix(dvec, nrow = nrow(x), ncol = nrow(y), byrow = F)
}
```

Data simulator

```
fnc.create.SMR.data <- function (N = N, K.trap = K.trap, K.camera = K.camera,
                                 sigma = sigma, g0trap = g0trap, lam0 = lam0,
                                 n.trap = n.trap, n.camera = n.camera,
                                 trap.design = trap.design,
                                 xlims = xlims, ylims = ylims,
                                 xlim.R = xlim.R, ylim.R = ylim.R,
                                 obsmod = c("pois", "bern"),
                                 n.collar = 10000, nlocs = 100,
                                 plot.map = TRUE, rnd = 2018) {
  # N = True number of animals in the state-space
  # K.trap = number of marking (trapping) occasions
  # K = number of resighting (eg camera) occasions
  # sigma = scale parameter for home range size
  # g0trap = probability of detection at the home range center for marking
  # lam0 = encounter rate at home range center for resighting
  # n.trap = total number of traps for marking.  Used to create a square
  #     grid of traps
  # n.camera = total number of cameras for resighting.  Used to create a square
  #     grid of detectors
  # trap.design = one of 'linear', 'grid', 'random'.
  # xlims = min and max coordinates of easting for the state-space
  # ylims = min and max coordinates of northing for the state-space
  # xlim.R = min and max coordinates of easting for the study area
  # ylim.R = min and max coordinates of northing for the study area.
  # obsmod = type of observation model for the resighting process.  We used
  #     'pois' for the manuscript.
  # n.collar = maximum number of animals to fit with telemetry tags or collars.
  #     Telemetry data is created for the lesser of n.collar or n.marked
  # nlocs = number of telemetry locations for each marked animal.  We used 100
```

```
#     for each marked animal in the manuscript

# VALUES RETURNED
# y.trap.all = all capture observations including known animals that were
#     undetected.  Array of individuals x trap x trap.occasion
# y.trap.marked = SCR observations of captured animals.  Array of
#     individuals x trap x trap.occasion
# y.camera.all = all resight observations including animals not detected
#     and/or not trapped.  Array of individuals x detector x occasion
# y.camera.marked = resight encounter history of marked animals.  Array of
#     individuals x detector x occasion
# n.camera.unmarked = number of detections of unmarked animals at location
#     j (row) and occasion k (column)
# i.marked = row numbers of marked animals in y.trap.all and y.camera.all
# i.unmarked = row numbers of unmarked animals in y.camera.all
# telemetry.array = Telemetry locations of marked individuals.  Array of
#     individual x location number x coordinates(x,y).
# X.trap = locations of traps for marking.  Matrix of trap x coordinates
# X.camera = locations of detectors for resighting.  Matrix of detector x
#     coordinates

set.seed(rnd)

if ( ! trap.design %in% c('grid', 'linear', 'random')){
  stop('trap.design should be one of:  grid, linear, or random')
}

obsmod <- match.arg(obsmod)
n.trap.row <- sqrt(n.trap)
n.camera.row <- sqrt(n.camera)
if (trap.design == 'grid'){
  coor0 <- seq(xlim.R[1], xlim.R[2], length = sqrt(n.trap))
  # Nets for a square grid of traps
  X.trap <- cbind(rep(coor0, each=length(coor0)),rep(coor0,
    times=length(coor0)))
}

if (trap.design == 'linear'){
  coor0 <- seq(xlim.R[1], xlim.R[2], length = n.trap)
  X.trap <- cbind(coor0, 0.5)
}

if (trap.design == 'random') {
  n.rand <- n.trap
  X.trap <- cbind( runif(n.rand, xlims[1], xlims[2]),
                   runif(n.rand, xlims[1], xlims[2]))
}
J.trap <- nrow(X.trap)  # nN

## Camera coordinates
coor0 <- seq(xlim.R[1], xlim.R[2], length = n.camera.row)
# Nets for a square grid of traps
X.camera <- cbind(rep(coor0, each=length(coor0)),rep(coor0,
  times=length(coor0)))
t.jitter <- (coor0[2] - coor0[1])/3
J.camera <- nrow(X.camera)  # nN

  # Activity Centers
  sx <- runif(N, xlims[1], xlims[2])
  sy <- runif(N, ylims[1], ylims[2])
  S <- cbind(sx, sy)
  #### MARK DATA
  D.trap <- e2dist(S, X.trap)
```

```
ptrap <- g0trap * exp(-(D.trap * D.trap)/(2 * sigma * sigma))
y.trap.all <-array(0, c(N, J.trap, K.trap))
for (i in 1:N){
  for (j in 1:J.trap){
    y.trap.all[i, j, ] <- rbinom(K.trap, 1, ptrap[i, j])
  }
}
n.trap.ind <- apply(y.trap.all, 1, sum)# number of captures per individual
marked <- ifelse(n.trap.ind > 0, 1, 0) # is each animal marked (0 or 1)
i.marked = (1:N)[marked == 1]          # ID for marked individuals
n.marked <- sum(marked)                # number captured and marked
y.trap <- y.trap.all[marked == 1, , ]  # capture-recapture data for marked ind.

#### RESIGHT DATA
# Distance between each home range center (row) and camera (column)
D <- e2dist(S, X.camera)
# Encounter rates
lam <- lam0 * exp(-(D * D)/(2 * sigma * sigma))
# Array for resighting data
y.camera.all <- array(NA, c(N, J.camera, K.camera))
for (i in 1:N) {
  for (j in 1:J.camera) {
    if (identical(obsmod, "bern")) {
      y.camera.all[i, j, ] <- rbinom(K.camera, 1, lam[i, j])
    }
    else if (identical(obsmod, "pois")) {
      y.camera.all[i, j, ] <- rpois(K.camera, lam[i, j])
    }
  }
}
y.camera.unmarked <- y.camera.all * (1 - marked)
i.unmarked <- (1:N)[rowSums(y.camera.unmarked) > 0]
# Sum of detections by Camera (row) and Occasion (column)
n.camera.unmarked <- apply(y.camera.unmarked, c(2, 3), sum)
# Resight data of marked animals
y.camera.marked <- y.camera.all[marked == 1, , ]
# Number of detections per individual
n.ind <- apply(y.camera.all, c(1), sum)
# was each individual detected by camera. Used in plot below.
det.camera <- ifelse(n.ind > 0, 1, 0)

# Telemetry data
n.collar <- min(c(n.marked, n.collar))
telemetry.array <- array(NA, dim=c(n.collar, nlocs, 2))
if (nlocs > 0 & n.collar > 0) {
  for (i in 1:n.collar) {
    telemetry.array[i, , 1] <- rnorm(nlocs, S[i.marked[i], 1], sigma)
    telemetry.array[i, , 2] <- rnorm(nlocs, S[i.marked[i], 2], sigma)
  }
}

# Plot marked and unmarked animals
if (plot.map == TRUE){
  par(mfrow = c(1,1))
  plot(S, col = 'red', pch = 19, xlim = xlims, ylim = ylims, asp = 1,
       xlab = 'X', ylab = 'Y',
       main = paste('Spatial Mark-Resight Simulated Data \n g0trap =', g0trap,
                    ' lam0camera =', lam0, ' sigma =', sigma, ' N =', N,
                    ' \n Trap Design:', trap.design,  sep = ' '))
  points(S, col = 'red', pch = 19, cex=1.75)
  points(X.trap, col='black', pch = 17, cex = 1.2)
  points(X.camera, col = 'black', pch = '+', cex = 0.8)
  points(S[det.camera == 1, 1], S[det.camera == 1, 2], pch=19, col='green',
```

```
        cex = 1.15)
      points(S[marked == 1, 1], S[marked == 1, 2], pch=19, col='purple', cex = 0.85)
      legend('topright', cex = 0.8,
        legend = c( 'Traps for Marking', 'Cameras', 'Home Range Centre',
        '  Marked', '  Detected Camera'),
        pch = c(17, 3, 19, 19, 19), pt.cex = c(1.2, 0.8, 1.75, 0.85, 1.15),
        col = c('black','black', 'red','purple', 'green' ),
        bg = 'gray95')
    } # End of Plot
    # Collect Results
    list(y.trap.all = y.trap.all, y.trap = y.trap, i.marked = i.marked,
         y.camera.all = y.camera.all, y.camera.marked = y.camera.marked,
         i.unmarked = i.unmarked,
         n.camera.unmarked = n.camera.unmarked,
         telemetry.array = telemetry.array, X.trap = X.trap, X.camera = X.camera)

}   # END OF FUNCTION  fnc.create.SMR.data
```

Auxiliary code to make *nnid* for use in the main code

```
# code to write out latent values that you can paste into model below for
# updating unidentified encounter histories.
# m: marked individuals (in the simulation, m=5)
# We can use sum or dsum
m<-5
paste0('nnid[j,k] ~ dsum(', paste0('y.unidentified[', 1:m, ',j,k]', collapse = ','),')')
```

Data augmentation

```
M <- 150
```

BUGS model

```
cat(file = "model.txt",
"
model {
  lam0.mark ~ dunif(0,1)     # Baseline encounter rate for marking occasions
  lam0.resight ~ dunif(0,2) # Resighting occasions
  sigma ~ dunif(0,2)
  sigma2 <- sigma^2
  psi ~ dbeta(1,1)
  id.prob ~ dunif(0,1)

  for(i in 1:M) {
    z[i] ~ dbern(psi)
    s[i,1] ~ dunif(xlim[1], xlim[2])
    s[i,2] ~ dunif(ylim[1], ylim[2])

    # Marking process
    for(j in 1:J.mark) {
      d.mark2[i,j] <- (s[i,1]-x.mark[j,1])^2 + (s[i,2]-x.mark[j,2])^2
      lambda.mark[i,j] <- lam0.mark*exp(-d.mark2[i,j]/(2*sigma2))*z[i]
      # Encounter histories from capture process for all individuals
      # including augmented population.
      y.mark[i,j] ~ dbinom(lambda.mark[i,j], K.mark)
    }

    for(j in 1:J.resight) {
      d.resight2[i,j] <- (s[i,1]-x.resight[j,1])^2 + (s[i,2]-x.resight[j,2])^2
```

```
      lambda.resight[i,j] <- lam0.resight * exp(-d.resight2[i,j]/(2*sigma2))*z[i]
    }
  }

  # Marked animals
  for(i in 1:n.marked)  {
    for(j in 1:J.resight) {
      for(k in 1:K.resight) {
        # Model for complete capture histories from marked animals
        y.full[i,j,k] ~ dpois(lambda.resight[i,j])
        # Model for observed capture histories from marked and identified
        y.obs[i,j,k] ~ dbin(id.prob, y.full[i,j,k])
        # Latent information from marked and unidentified individuals
        y.unidentified[i,j,k]<- (y.full[i,j,k]- y.obs[i,j,k])
      }
    }
  }

  for(j in 1:J.resight){
    for(k in 1:K.resight){
      # It can be used the newer sum distribution
      nnid[j,k] ~ dsum(y.unidentified[1,j,k], y.unidentified[2,j,k], y.unidentified[3,j,k],
                       y.unidentified[4,j,k], y.unidentified[5,j,k])
    }
  }


  # Resight likelihoods of unmarked animals
  for(j in 1:J.resight){
    bigLambda[j] <- sum(lambda.resight[(n.marked+1):M,j])
    for(k in 1:K.resight){
      n.unmarked[j,k] ~ dpois(bigLambda[j])  # 2D detections of uncollared individuals
    }
  }

  # Telemetry data for n.collared animals
  for (i in 1:n.collar){
    for (j in 1:n.locs){
      telemetry.array[i, j, 1] ~ dnorm(s[i, 1], 1 / sigma2)
      telemetry.array[i, j, 2] ~ dnorm(s[i, 2], 1 / sigma2)
    }
  }

  N <- sum(z[1:M])
}
")
```

Data simulation

```
N<-50

df <- fnc.create.SMR.data(N = N,
                          K.trap = 5,
                          K.camera = 4,
                          sigma = 0.05,
                          g0trap = 0.05,
                          lam0 = 0.5,
                          n.trap = 25,
                          n.camera = 100,
                          trap.design = 'grid',
                          xlims = 0:1, ylims = 0:1,
```

```
                    xlim.R = c(0.2, 0.8), ylim.R = c(0.2, 0.8),
                    obsmod = "pois",
                    n.collar = 5,
                    nlocs = 100,
                    rnd= 334)
```

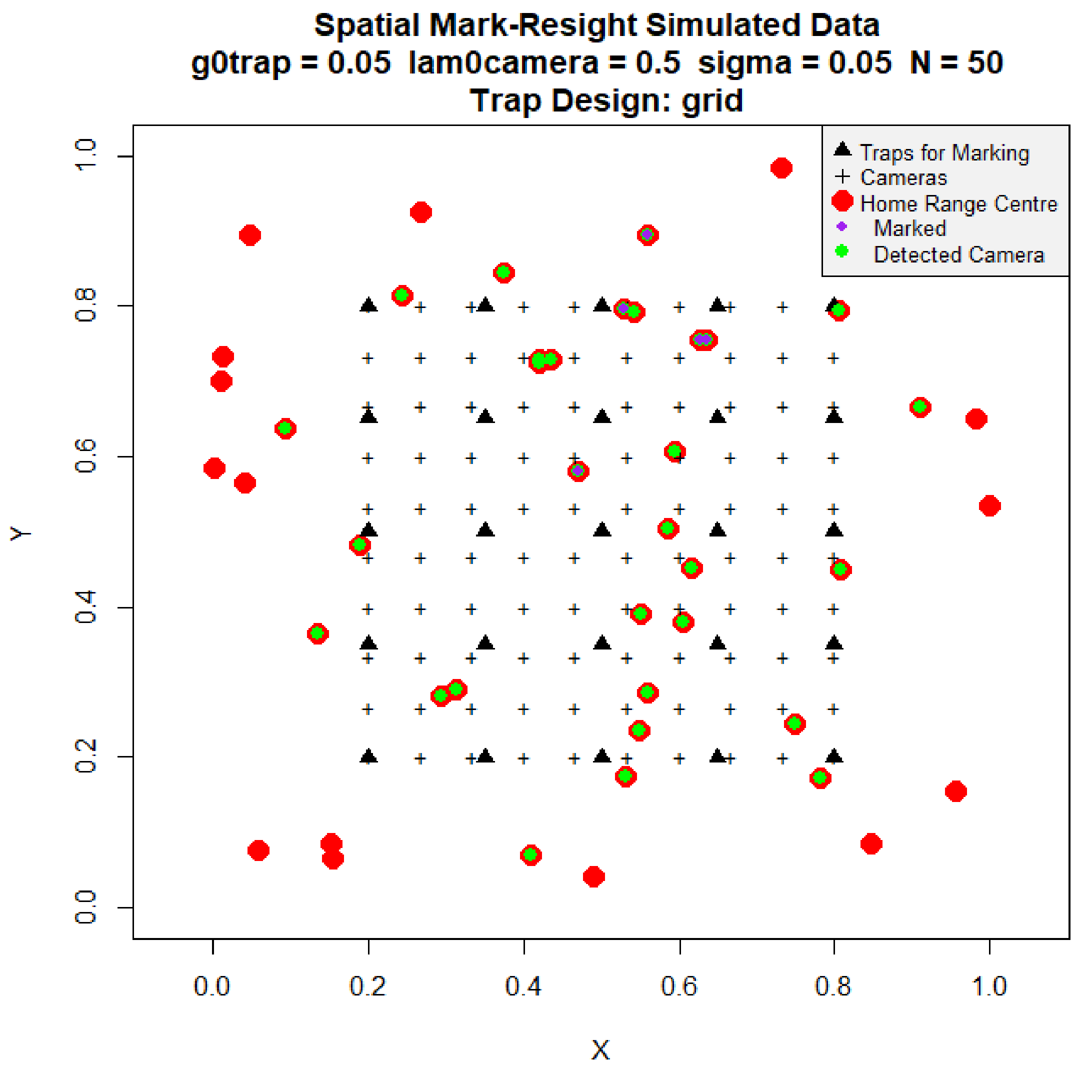

Bundle and summarize data set

```
n.marked <- length(df$i.marked)
M <- 150
marked <- rep(0, M)
marked[1:n.marked] <- 1
y.trap.mx <- apply(df$y.trap, c(1, 2), sum)     # sum detections across occasions
y.trap.aug <- matrix(0, nrow = M, ncol = nrow(df$X.trap))
y.trap.aug[1:n.marked, ] <- y.trap.mx

y.rs.aug <- array(NA, c(M, nrow(df$X.camera), dim(df$y.camera.marked)[3]))
y.rs.aug[1:n.marked, , ] <- df$y.camera.marked
y.rs.marked <- array(0, c(M, nrow(df$X.camera), dim(df$y.camera.marked)[3]))
y.rs.marked[1:n.marked, , ] <- df$y.camera.marked

J.resight <- nrow(df$X.camera)
```

```
K.resight <- dim(y.rs.aug)[3]
y.id2<-df$y.camera.marked; sum(y.id2)
## [1] 30
```

Fixing identification rate

```
id.prob<-0.0 ############# FIXING THE IDENTIFICATION RATE ######################

y.id<-array(0,c(n.marked,J.resight, K.resight))
for(i in 1:n.marked){
  for(j in 1:J.resight){
    for(k in 1:K.resight){
      y.id[i,j,k]<-rbinom(prob=id.prob, 1, y.id2[i,j,k])
    }
  }
}

sum(y.id)
## [1] 0  # None identified
```

Prepare data (cont.)

```
ycam.rec<-apply(y.id,c(2,3),sum)
nnid<-apply(df$y.camera.marked, c(2,3), sum) - ycam.rec    # Total unidentified events
sum(y.id)/sum(df$X.camera)
## [1] 0
```

Data

```
data  <-  list (x.mark = df$X.trap,           # live traps
       y.mark = y.trap.aug,                   # y.mark: live trap records
       x.resight = df$X.camera,               # camera traps
       y.obs = y.id,                          # recaptures records from
                                              #   marked-recognizable individuals
       nnid=nnid,                             # events from marked and unidentified
                                              #   individuals
       n.unmarked = df$n.camera.unmarked,     # events from unmarked individuals
       telemetry.array = df$telemetry.array,  # telemetry data
       xlim = c(0,1), ylim = c(0,1),
       M=M,
       n.marked = n.marked,                   # number if marked individuals
       K.mark = dim(df$y.trap)[3],            # occasions of live trap
       J.mark = nrow(df$X.trap),              # number of live-trap
       K.resight = dim(y.rs.aug)[3],          # occasions of resight in cameras
       J.resight = nrow(df$X.camera),         # number of cameras
       n.collar = dim(df$telemetry.array)[1],# number of telemetry tagged ind.
       n.locs = dim(df$telemetry.array)[2])   # number of telemetry data
str(data)
## List of 17
##  $ x.mark         : num [1:25, 1:2] 0.2 0.2 0.2 0.2 0.2 0.35 0.35 0.35 0.35 0.35 ...
##  $ y.mark         : num [1:150, 1:25] 0 0 0 0 0 0 0 0 0 0 ...
##  $ x.resight      : num [1:100, 1:2] 0.2 0.2 0.2 0.2 0.2 0.2 0.2 0.2 0.2 0.2 ...
##  $ y.obs          : num [1:5, 1:100, 1:4] 0 0 0 0 0 0 0 0 0 0 ...
##  $ nnid           : num [1:100, 1:4] 0 0 0 0 0 0 0 0 0 0 ...
##  $ n.unmarked     : num [1:100, 1:4] 0 0 0 0 0 1 0 0 0 0 ...
##  $ telemetry.array: num [1:5, 1:100, 1:2] 0.699 0.532 0.459 0.607 0.522 ...
##  $ xlim           : num [1:2] 0 1
##  $ ylim           : num [1:2] 0 1
##  $ M              : num 150
```

```
##  $ n.marked       : int 5
##  $ K.mark         : int 5
##  $ J.mark         : int 25
##  $ K.resight      : int 4
##  $ J.resight      : int 100
##  $ n.collar       : int 5
##  $ n.locs         : int 100
```

Setting inits

```
# Generate starting values and latent encounter history
zst <- rbinom(M, 1, 0.4)
zst[1:n.marked] <- 1
s.start <- cbind(runif(M, 0, 1), runif(M, 0, 1))
s.start[1:n.marked, 1] <- rowMeans(df$telemetry.array[ , ,1])
s.start[1:n.marked, 2] <- rowMeans(df$telemetry.array[ , ,2])

# We need to carefully tailor the inits for y.unidentified
d <- e2dist(s.start[1:n.marked,], df$X.camera)
lam <- 0.5 * exp( -(d^2)/(2 * 0.05^2))
yi <- array(0, c(n.marked, J.resight, K.resight)) # resighting array
for (j in 1:J.resight) {
  for (k in 1:K.resight) {
    if (nnid[j, k] > 0) {
      probs <- lam[ ,j]
      probs <- probs / sum(probs)
      latent.id <- sample(1:n.marked, nnid[j,k], prob = probs, replace = FALSE)
      yi[latent.id , j, k] <- 1
    }
  } # end of k
}   # end of j

yi <- yi + y.id
```

Inits

```
inits<-function(){list(z = zst,      # Data augmentation
       s=s.start,                    # state-space sampler
       y.full=yi,                    # init for latent marked individual record
       lam0.mark=0.05,               # baseline rate for live-trap
       lam0.resight = 0.7,           # baseline rate for resight in cameras
       sigma=0.05,                   # init for half-normal parameter
       psi = 0.25)}

str(inits())
## List of 7
##  $ z           : num [1:150] 1 1 1 1 1 0 0 0 0 0 ...
##  $ s           : num [1:150, 1:2] 0.63 0.528 0.469 0.632 0.557 ...
##  $ y.full      : num [1:5, 1:100, 1:4] 0 0 0 0 0 0 0 0 0 0 ...
##  $ lam0.mark   : num 0.05
##  $ lam0.resight: num 0.7
##  $ sigma       : num 0.05
##  $ psi         : num 0.25
```

Parameters to save

```
params<-c('lam0.mark', 'lam0.resight', 'sigma', 'psi', 'N', 'id.prob')
```

Run JAGS

```
out <- jags(data = data, inits = inits, params, 'model.txt',
  n.chains = 3, n.adapt = 1000, n.iter = 5000, n.burnin = 1000, n.thin = 1,
  parallel = FALSE)
##
## Processing function input.......
##
## Done.
##
## Compiling model graph
##    Resolving undeclared variables
##    Allocating nodes
## Graph information:
##    Observed stochastic nodes: 7550
##    Unobserved stochastic nodes: 2455
##    Total graph size: 111625
##
## Initializing model
##
## Adaptive phase, 1000 iterations x 3 chains
## If no progress bar appears JAGS has decided not to adapt
##
##
##  Burn-in phase, 1000 iterations x 3 chains
##
##
## Sampling from joint posterior, 4000 iterations x 3 chains
##
##
## Calculating statistics.......
##
## Done.
print(out,dig=3)
## JAGS output for model 'model.txt', generated by jagsUI.
## Estimates based on 3 chains of 5000 iterations,
## adaptation = 1000 iterations (sufficient),
## burn-in = 1000 iterations and thin rate = 1,
## yielding 12000 total samples from the joint posterior.
## MCMC ran for 184.531 minutes at time 2018-10-16 18:18:28.
##
##                    mean     sd      2.5%       50%     97.5% overlap0 f  Rhat n.eff
## lam0.mark         0.066  0.029     0.023     0.062     0.134    FALSE 1 1.000 12000
## lam0.resight      0.592  0.094     0.425     0.586     0.789    FALSE 1 1.000  1750
## sigma             0.050  0.001     0.048     0.050     0.053    FALSE 1 1.000  6795
## psi               0.333  0.070     0.209     0.328     0.479    FALSE 1 1.003   780
## N                49.546  8.938    34.000    49.000    69.000    FALSE 1 1.005   602
## id.prob           0.031  0.031     0.001     0.022     0.111    FALSE 1 1.000  9335
## deviance      -2636.128 11.679 -2657.212 -2636.739 -2612.149    FALSE 1 1.000 10269
##
##
## Successful convergence based on Rhat values (all < 1.1).
## Rhat is the potential scale reduction factor (at convergence, Rhat=1).
## For each parameter, n.eff is a crude measure of effective sample size.
##
## overlap0 checks if 0 falls in the parameter's 95% credible interval.
## f is the proportion of the posterior with the same sign as the mean;
## i.e., our confidence that the parameter is positive or negative.
##
## DIC info: (pD = var(deviance)/2)
## pD = 68.2 and DIC = -2567.927
## DIC is an estimate of expected predictive error (lower is better).
```
