# Supplementary material for: Generalized spatial mark–resight models with incomplete identification: An application to red fox density estimates
Source: Ecol Evol. 2019 Mar 22;9(8):4739–48. doi: 10.1002/ece3.5077 (PMC6476752; doi:10.1002/ece3.5077)
Supplement: Supplementary file 3 [file ECE3-9-4739-s003.pdf]

Supporting Information S3: Results of movement parameter σ in Gen-SMR-ID simulations.

Generalized Spatial Mark-Resight models with incomplete identification: an application to red fox density estimates

José Jiménez[1], Richard Chandler[2], Jorge Tobajas[1], Esther Descalzo[1], Rafael Mateo[1], Pablo Ferreras[1]

[1]Instituto de Investigación en Recursos Cinegéticos (IREC, CSIC-UCLM-JCCM), Ronda de Toledo 12, 13071 Ciudad Real, Spain.
[2]University of Georgia, Warnell School of Forestry and Natural Resources.

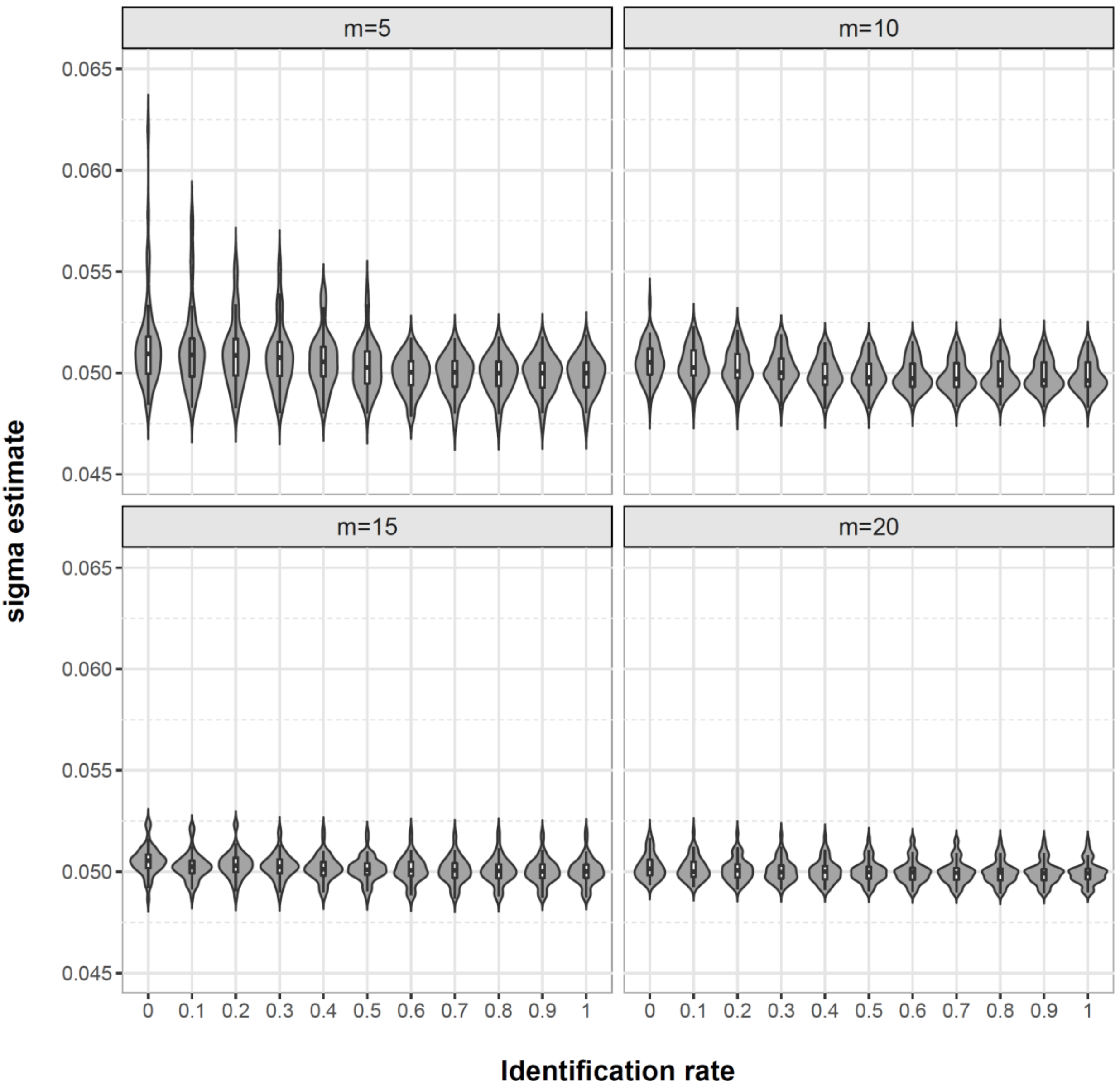

Figure 1. Posterior means violin plot of movement parameter (σ) estimates using Generalized Spatial Mark-Resight with incomplete identification model (Gen-SMR-ID) in JAGS from different numbers of marked $m \in \{5, 10, 15, 20\}$ individuals, plotted against the rate of identification (ID).
