# Supplementary material for: Generalized spatial mark–resight models with incomplete identification: An application to red fox density estimates
Source: Ecol Evol. 2019 Mar 22;9(8):4739–48. doi: 10.1002/ece3.5077 (PMC6476752; doi:10.1002/ece3.5077)
Supplement: Supplementary file 4 [file ECE3-9-4739-s004.pdf]

Supporting Information S4: Convergence Diagnostics

Generalized Spatial Mark-Resight models with incomplete identification: an application to red fox density estimates

José Jiménez[1], Richard Chandler[2], Jorge Tobajas[1], Esther Descalzo[1], Rafael Mateo[1], Pablo Ferreras[1]

[1]Instituto de Investigación en Recursos Cinegéticos (IREC, CSIC-UCLM-JCCM), Ronda de Toledo 12, 13071 Ciudad Real, Spain.
[2]University of Georgia, Warnell School of Forestry and Natural Resources.

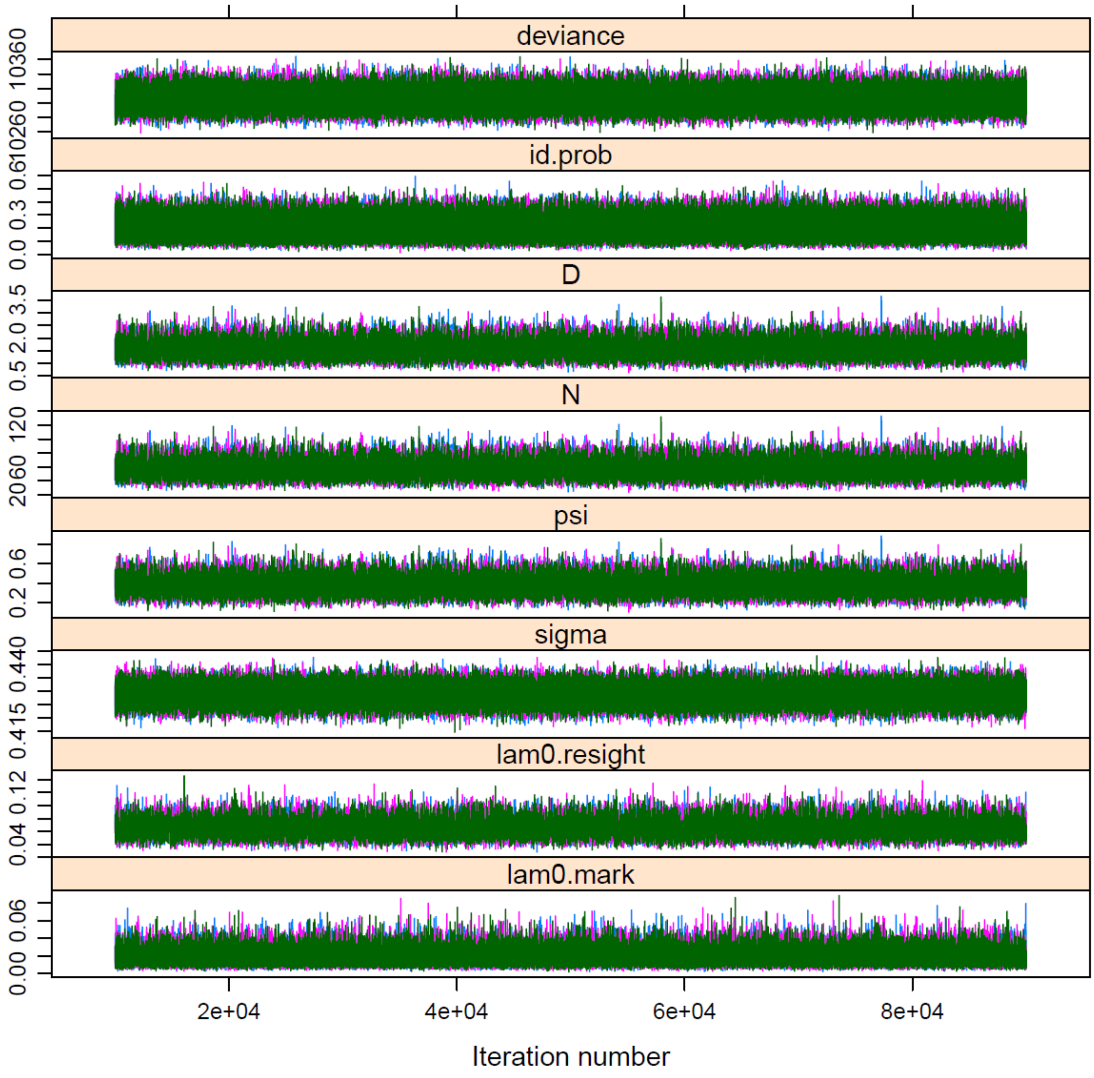

Figure 1. La Nava. MCMC trace plots for the three Markov chains, 85000 iterations, discarding 5000 burn-in iterations, yielding 240000 total samples

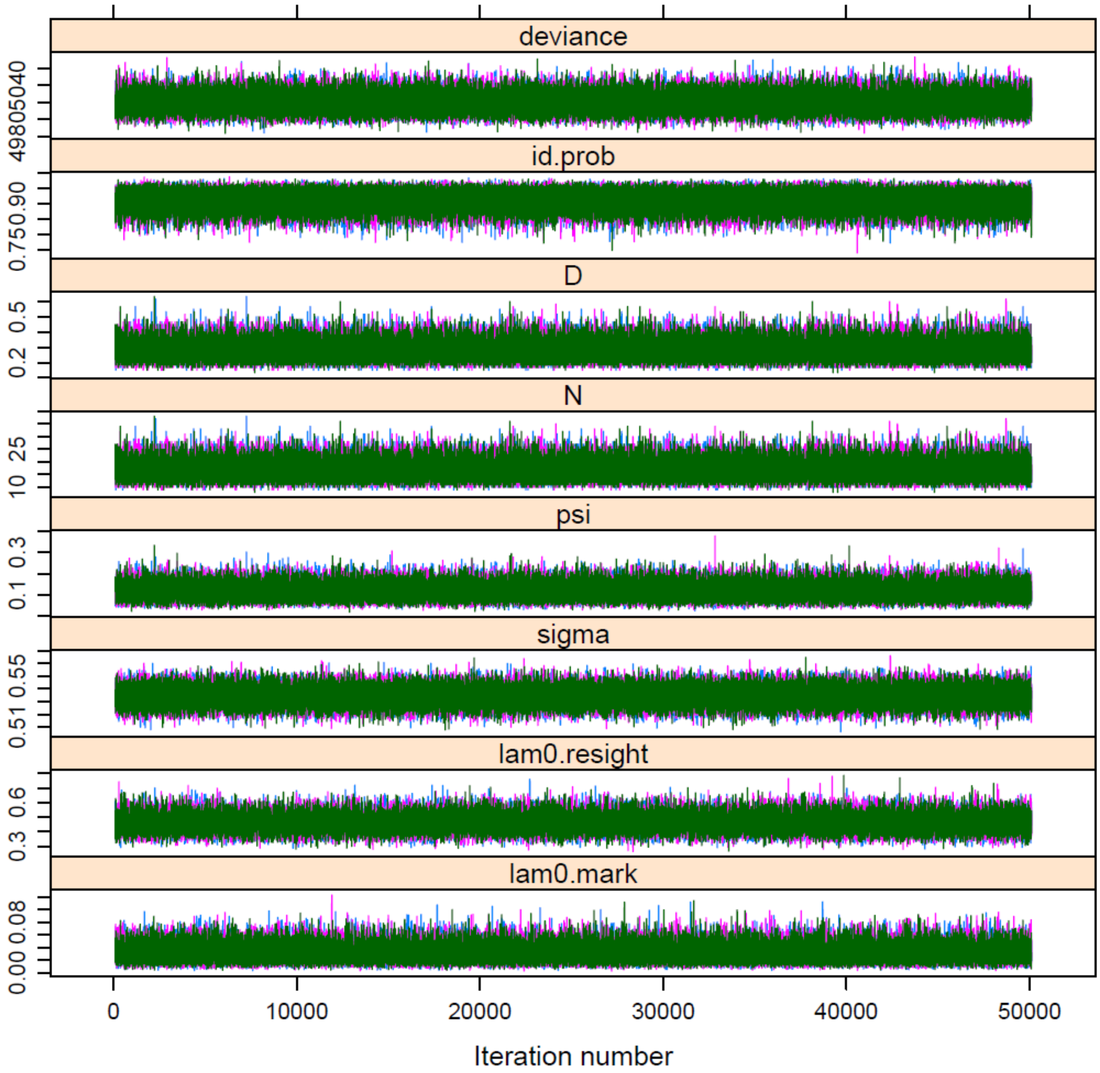

Figure 2. Los Pilones. MCMC trace plots for the three Markov chains, 52500 iterations, discarding 2500 burn-in iterations, yielding 150000 total samples.
