# Supplementary material for: Generalized spatial mark–resight models with incomplete identification: An application to red fox density estimates
Source: Ecol Evol. 2019 Mar 22;9(8):4739–48. doi: 10.1002/ece3.5077 (PMC6476752; doi:10.1002/ece3.5077)
Supplement: Supplementary file 5 [file ECE3-9-4739-s005.pdf]

Supporting Information S5: Model outputs from Gen-SMR-ID in La Nava and Los Pilones for all marked individuals unidentified

Generalized Spatial Mark-Resight models with incomplete identification: an application to red fox density estimates

José Jiménez[1], Richard Chandler[2], Jorge Tobajas[1], Esther Descalzo[1], Rafael Mateo[1], Pablo Ferreras[1]

[1]Instituto de Investigación en Recursos Cinegéticos (IREC, CSIC-UCLM-JCCM), Ronda de Toledo 12, 13071 Ciudad Real, Spain.
[2]University of Georgia, Warnell School of Forestry and Natural Resources.

Table 1. Posterior mean, standard deviation and 95% HPD interval coverage of model parameters from the Gen-SMR-ID model from red fox (*Vulpes vulpes*) study cases in La Nava and Los Pilones (Ciudad Real, Central Spain). We discarded from the model all information about individual identification. Baseline capture rate ($\lambda_{0.mark}$), baseline resighting rate ($\lambda_{0.resight}$), parameter of movement (σ), data augmentation parameter (ψ), population size estimate in the state space ($\widehat{N}$), density estimate ($\widehat{D}$), posterior probability of identification (δ) and deviance.

La Nava

	mean	sd	2.50%	50%	97.50%
lam0.mark ($\lambda_{0.mark}$)	0.02	0.01	0.01	0.02	0.04
lam0.resight ($\lambda_{0.resight}$)	0.07	0.02	0.04	0.06	0.11
sigma (σ)	0.43	0.00	0.42	0.43	0.44
psi (ψ)	0.40	0.10	0.23	0.39	0.61
$\widehat{N}$	59.58	13.51	37.00	58.00	89.00
$\widehat{D}$	1.64	0.37	1.02	1.59	2.44
δ	0.03	0.03	0.00	0.02	0.11
deviance	10323.40	13.51	10298.19	10322.98	10351.21

Los Pilones

	mean	sd	2.50%	50%	97.50%
lam0.mark ($\lambda_{0.mark}$)	0.04	0.02	0.02	0.04	0.08
lam0.resight ($\lambda_{0.resight}$)	0.59	0.09	0.43	0.58	0.79
sigma (σ)	0.55	0.01	0.53	0.55	0.56
psi (ψ)	0.11	0.03	0.06	0.11	0.18
$\widehat{N}$	16.01	3.10	11.00	16.00	23.00
$\widehat{D}$	0.27	0.05	0.18	0.27	0.39
δ	0.01	0.01	0.00	0.01	0.03
deviance	5073.38	11.95	5051.26	5072.93	5098.11

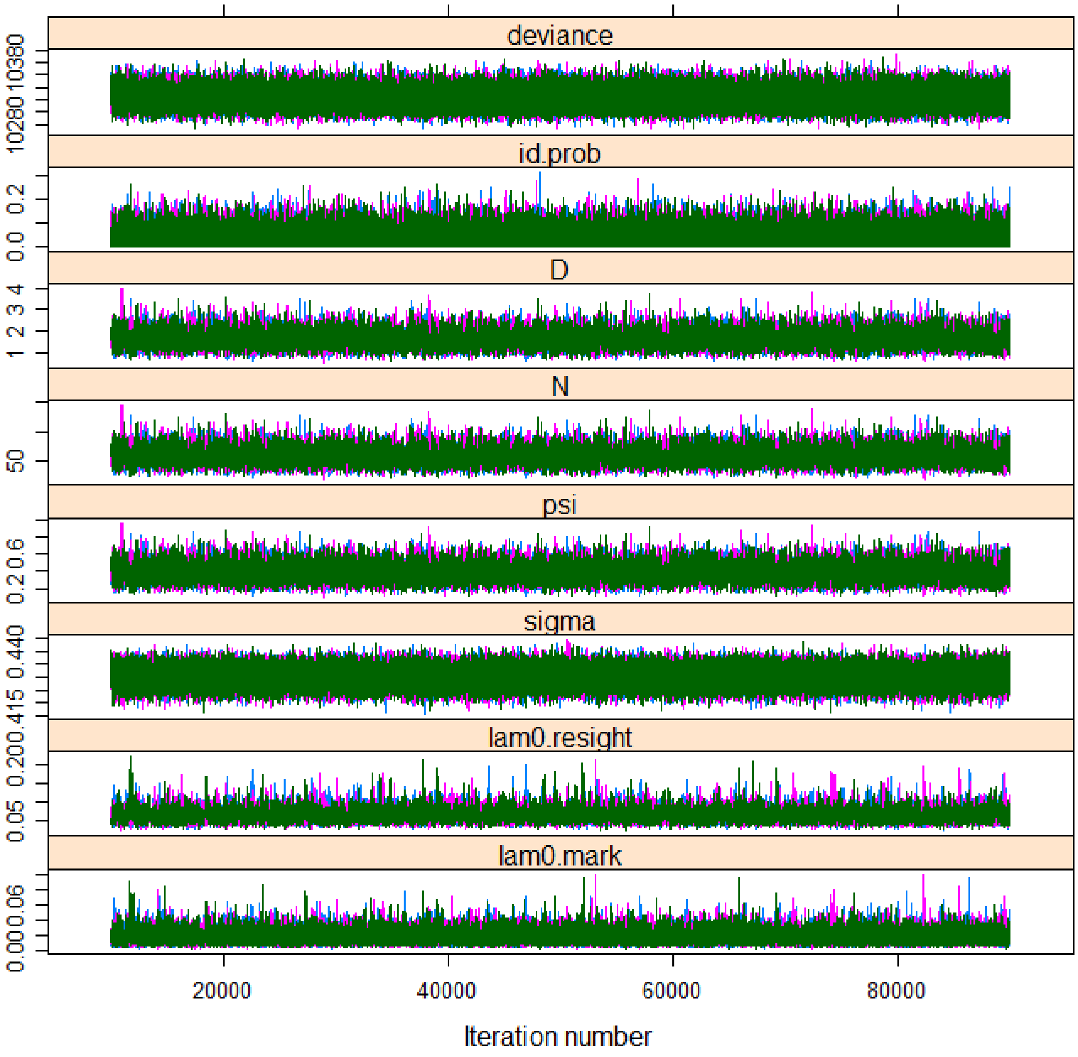

Figure 1. La Nava. MCMC trace plots for the three Markov chains, 85000 iterations, discarding 5000 burn-in iterations, yielding 240000 total samples.

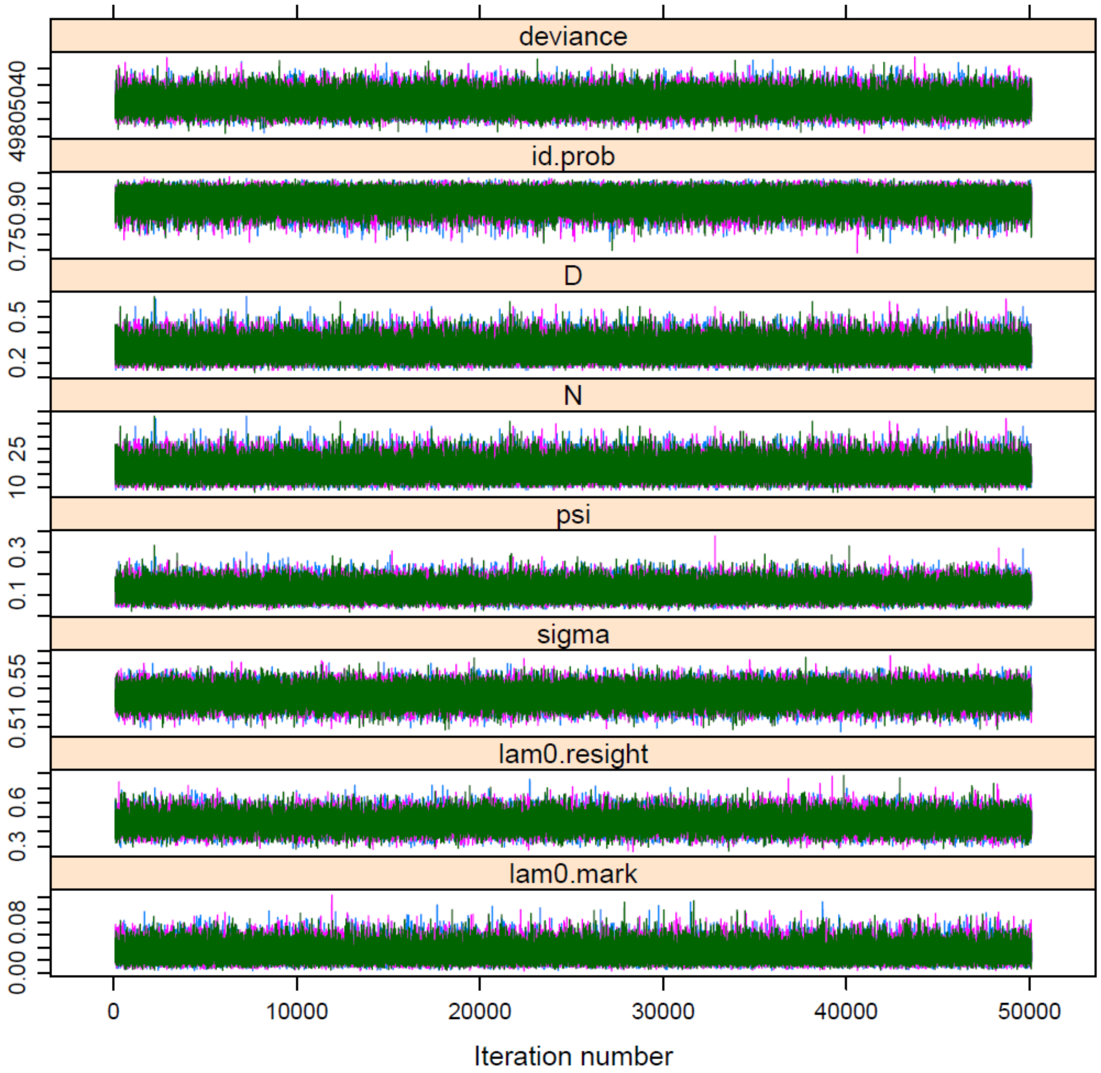

Figure 2. Los Pilones. MCMC trace plots for the three Markov chains, 52500 iterations, discarding 2500 burn-in iterations, yielding 150000 total samples.
