# Supplementary material for: Generalized spatial mark–resight models with incomplete identification: An application to red fox density estimates
Source: Ecol Evol. 2019 Mar 22;9(8):4739–48. doi: 10.1002/ece3.5077 (PMC6476752; doi:10.1002/ece3.5077)
Supplement: Supplementary file 6 [file ECE3-9-4739-s006.pdf]

Supporting Information S6: Fitting red fox density estimates (La Nava) using distance sampling. R + unmarked script

Generalized Spatial Mark-Resight models with incomplete identification: an application to red fox density estimates

José Jiménez[1], Richard Chandler[2], Jordi Tobajas[1], Esther Descalzo[1], Rafael Mateo[1], Pablo Ferreras[1]

[1]Instituto de Investigación en Recursos Cinegéticos (IREC, CSIC-UCLM-JCCM), Ronda de Toledo 12, 13071 Ciudad Real, Spain.
[2]University of Georgia, Warnell School of Forestry and Natural Resources.

Table of Contents

Define working directory 1
Data 2
Package into unmarked DS data frame 2
Models 2
Model selection 3
Results using halfnormal and Poisson 3
Goodness of fitting model (GoF) 4
Package into unmarked GDS data frame 6
Models 6
Model selection 6
Results using halfnormal and Negative Binomial 7
Goodness of fitting model (GoF) 7

Define working directory

```
setwd('C:/…')
library("unmarked")
## Loading required package: reshape
## Loading required package: lattice
## Loading required package: parallel
## Loading required package: Rcpp
```

Data

```
dat2<-read.table("FoxTransects.txt", header=TRUE)
dat2$visit<-as.factor(dat2$visit)
dat2$site<-as.factor(dat2$site)
transect.length <- read.table("LTran.txt", header=FALSE)
range(dat2$d)
## [1]  49.49 366.00
cutpt <- seq(0, 400, by=50) #create distance categories (m)
y2 <- formatDistData(dat2, "distance", "site", cutpt, "visit")

y2<-y2[order(as.numeric(rownames(y2))),,drop=FALSE]
```

Stacking data:

```
ystacked<- rbind(y2[,1:8], y2[,9:16], y2[,17:24], y2[,25:32], y2[,33:40])
```

Package into unmarked DS data frame

Create an unmarkedFrame and fit the models using *distsamp*

```
umf.stacked <- unmarkedFrameDS(y = ystacked, survey="line", unitsIn="m",
   dist.breaks=cutpt, tlength=c(transect.length[,1]),
   siteCovs = NULL)
summary(umf.stacked)
## unmarkedFrameDS Object
##
## line-transect survey design
## Distance class cutpoints (m):  0 50 100 150 200 250 300 350 400
##
## 15 sites
## Maximum number of distance classes per site: 8
## Mean number of distance classes per site: 8
## Sites with at least one detection: 11
##
## Tabulation of y observations:
##   0  1  2  3
## 94 18  7  1
```

Models

Fitting competing distance models

```
# half-normal detection function
fm.hn.p <- distsamp(~1 ~1,
   keyfun = "halfnorm", output = "density", unitsOut = "kmsq", data = umf.stacked)
# half-normal detection function
fm.exp <- distsamp(~1 ~1,  start=c(-4,30),
   keyfun = "exp", output = "density", unitsOut = "kmsq", data = umf.stacked)
# half-normal detection function
fm.hz <- distsamp(~1 ~1,   start=c(-4,20,-25),
   keyfun = "hazard", output = "density", unitsOut = "kmsq", data = umf.stacked)
```

Model selection

Create a fitList and a model selection table

```
f1 <- fitList('Halfnormal'    =fm.hn.p,
              'Exponential'   =fm.exp,
              'Hazard rate'   =fm.hz)

modSel(f1)
##             nPars    AIC delta AICwt cumltvWt
## Halfnormal      2 168.67  0.00 0.884     0.88
## Exponential     2 173.35  4.68 0.085     0.97
## Hazard rate     3 175.35  6.68 0.031     1.00
```

Results using halfnormal and Poisson

```
#Backtransformed results of the better model:
backTransform(fm.hn.p, type="state")
## Backtransformed linear combination(s) of Density estimate(s)
##
##  Estimate    SE LinComb (Intercept)
##      1.27 0.292   0.237           1
##
## Transformation: exp
```

View the frequency distribution for perpendicular distances as a final check

```
hist(fm.hn.p, lwd=3, col="lightgrey", ylim=c(0,.006), main="")
```

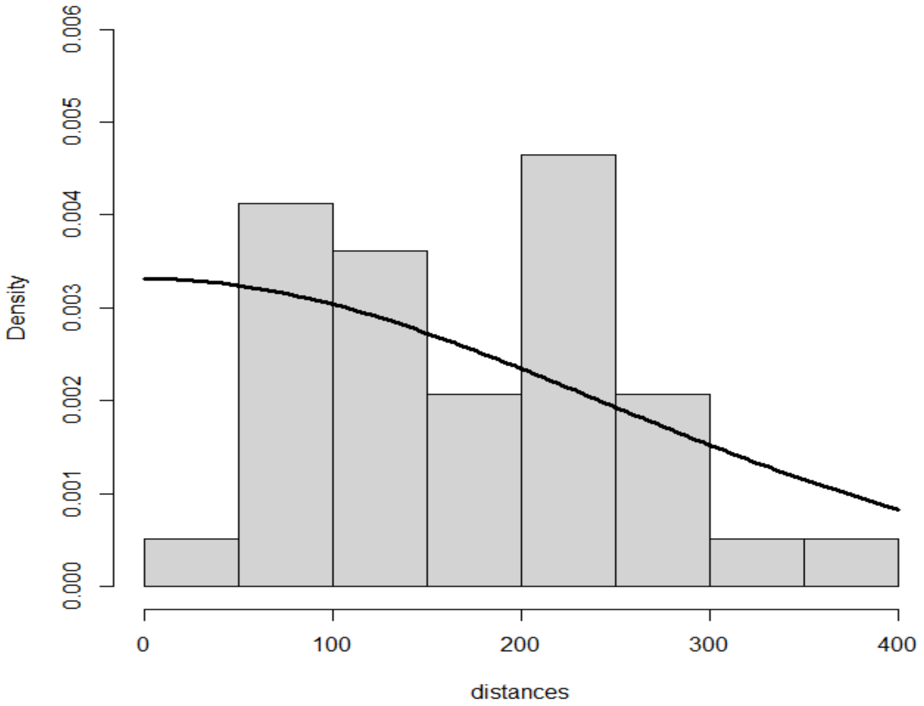

Goodness of fitting model (GoF)

Check out the goodness-of-fit of this model

```
fitstats <- function(fm) {
  observed <- getY(fm@data)
  expected <- fitted(fm, na.rm = TRUE)
  resids <- residuals(fm, na.rm = TRUE)
  sse <- sum(resids^2, na.rm = TRUE)
  chisq <- sum((observed - expected)^2 / expected, na.rm = TRUE)
  freeTuke <- sum((sqrt(observed) - sqrt(expected))^2, na.rm = TRUE)
  out <- c(SSE=sse, Chisq=chisq, freemanTukey=freeTuke)
  return(out)
}

(pb <- parboot(fm.exp, fitstats, nsim=10000, report=1))
## t0 = 45.18941 158.1229 37.72548
## Running in parallel.  Bootstrapped statistics not reported.
##
## Call:
## parboot(object = fm.exp, statistic = fitstats, nsim = 10000, report = 1)
##
## Parametric Bootstrap Statistics:
##                 t0 mean(t0 - t_B) StdDev(t0 - t_B) Pr(t_B > t0)
## SSE           45.2          10.57             7.68       0.0895
## Chisq        158.1          39.09            17.37       0.0258
## freemanTukey  37.7           4.07             3.63       0.1272
##
## t_B quantiles:
##              0% 2.5% 25% 50% 75% 97.5% 100%
## SSE          14   22  29  34  39    52   81
## Chisq        74   91 107 117 129   158  216
## freemanTukey 20   26  31  34  36    41   49
##
## t0 = Original statistic compuated from data
## t_B = Vector of bootstrap samples
```

Histograms of GoF

```
par(mfrow=c(2,2))
par(mar = c(5.00,
            5.00,
            2.00,
            1.00))
hist(pb@t.star[,1], xlab="SSE", col="lightgrey",
  font.lab=2, cex.lab=1, main="")
abline(v=pb@t0[1], lty=2, lwd=2)
par(mar = c(5.00,
            1.00,
            2.00,
            2.00))
hist(pb@t.star[,2], xlab="Chisq", col="lightgrey",
  font.lab=2, cex.lab=1, main="")
abline(v=pb@t0[2], lty=2, lwd=2)
par(mar = c(5.00,
            5.00,
            1.00,
            0.00))
hist(pb@t.star[,3], xlab="freemanTukey", col="lightgrey", breaks=15,
```

```
  font.lab=2, cex.lab=1, main="")
abline(v=pb@t0[3], lty=2, lwd=2)
```

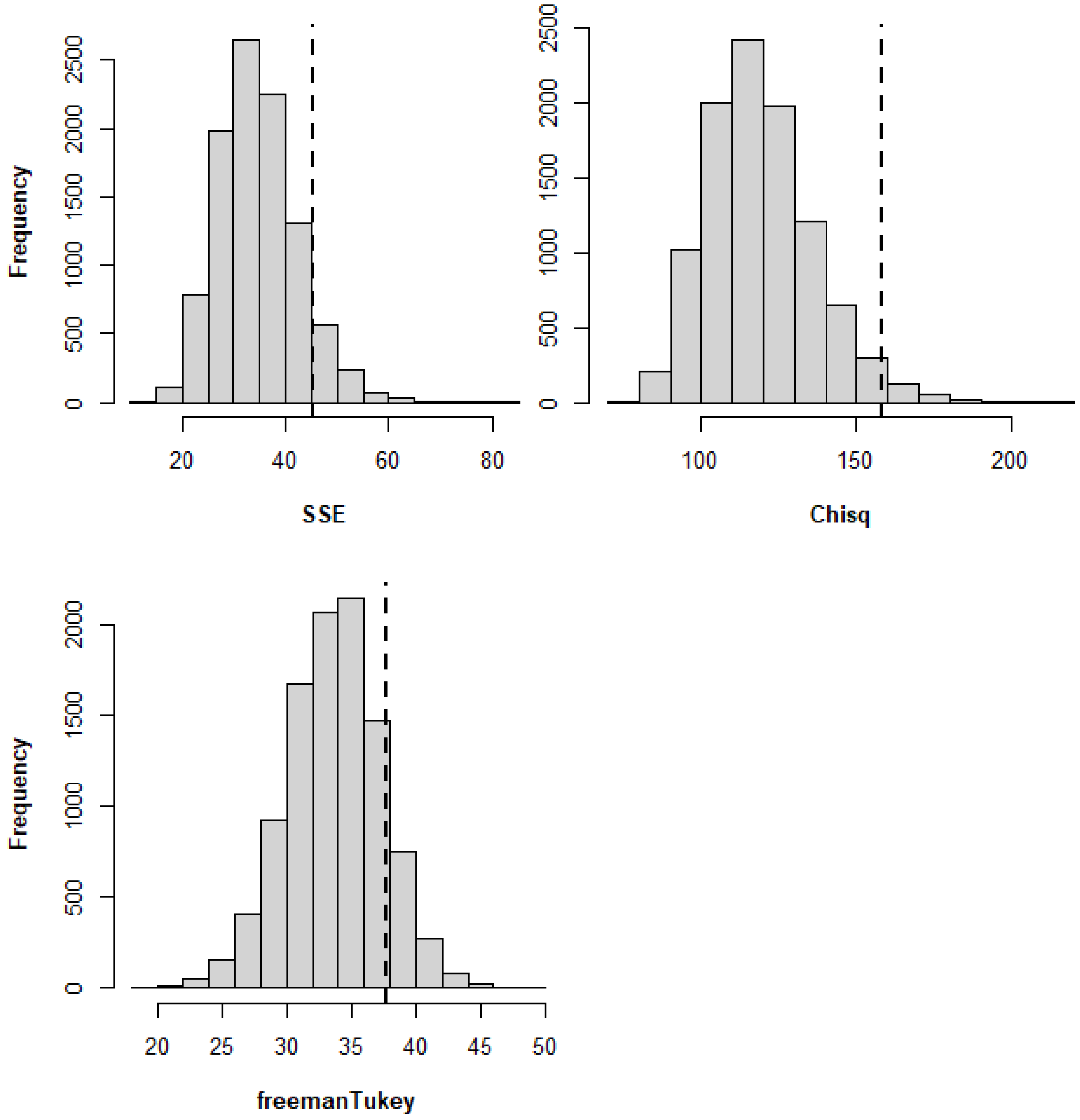

Chisquare test statistic

```
# c-hat as ratio of observed and mean of expected value of Chi2
(c.hat <- pb@t0[2] / mean(pb@t.star[,2]))
##    Chisq
## 1.328427
# Evidence of over-dispersion
```

Package into unmarked GDS data frame

Fit the model using *gdistsamp*

```
umf.stacked.v2 <- unmarkedFrameGDS(y = ystacked, survey="line", unitsIn="m",
   dist.breaks=cutpt, numPrimary=1, tlength=c(transect.length[,1]),
   siteCovs =NULL)
summary(umf.stacked.v2)
## unmarkedFrame Object
##
## 15 sites
## Maximum number of observations per site: 8
## Mean number of observations per site: 8
## Number of primary survey periods: 1
## Number of secondary survey periods: 1
## Sites with at least one detection: 11
##
## Tabulation of y observations:
##  0  1  2  3
## 94 18  7  1
```

Models

Fitting competing distance models

```
# half-normal-Poisson detection function
fm0.hn.p <- gdistsamp(~1, ~1, ~1,
   keyfun = "halfnorm", output = "density", unitsOut = "kmsq",
   mixture="P", data = umf.stacked.v2)
# half-normal-Negative Binomial detection function
fm0.hn.NB <- gdistsamp(~1, ~1, ~1,
   keyfun = "halfnorm", output = "density", unitsOut = "kmsq",
   mixture="NB", data = umf.stacked.v2)
# Exponential-Poisson detection function
fm0.exp.p <- gdistsamp(~1, ~1, ~1,
   keyfun = "exp", output = "density", unitsOut = "kmsq", mixture="P",
   data = umf.stacked.v2)
# Exponential-Negative Binomial detection function
fm0.exp.NB <- gdistsamp(~1, ~1, ~1,
   keyfun = "exp", output = "density", unitsOut = "kmsq", mixture="NB",
   data = umf.stacked.v2)
# Hazard-rate-Poisson detection function
fm0.hr.p <- gdistsamp(~1, ~1, ~1,  start=c(-5.5, 20,-20),
   keyfun = "hazard", output = "density", unitsOut = "kmsq", mixture="P",
   data = umf.stacked.v2)
# Hazard-rate-Negative Binomial detection function
fm0.hr.NB <- gdistsamp(~1, ~1, ~1, start=c(-4.2, 15,-20, 1),
   keyfun = "hazard", output = "density", unitsOut = "kmsq", mixture="NB",
   data = umf.stacked.v2)
```

Model selection

Create a fitList and a model selection table

```
f2 <- fitList('Half.-Pois. lambda(.)phi(.)det(.)'        =fm0.hn.p,
              'Half.-Neg. Bin, lambda(.)phi(.)det(.)'    =fm0.hn.NB,
```

```
              'Exp.-Pois. lambda(.)phi(.)det(.)'         =fm0.exp.p,
              'Exp.-Neg. Bin, lambda(.)phi(.)det(.)'     =fm0.exp.NB,
              'Haz. rate-Pois, lambda(.)phi(.)det(.)'    =fm0.hr.p,
              'Haz. rate-Neg. Bin. lambda(.)phi(.)det(.)'=fm0.hr.NB)
modSel(f2)
##                                           nPars    AIC delta AICwt
## Half.-Neg. Bin, lambda(.)phi(.)det(.)         3 154.60  0.00 0.456
## Half.-Pois. lambda(.)phi(.)det(.)             2 155.38  0.78 0.309
## Exp.-Neg. Bin, lambda(.)phi(.)det(.)          3 157.20  2.60 0.124
## Exp.-Pois. lambda(.)phi(.)det(.)              2 157.98  3.38 0.084
## Haz. rate-Neg. Bin. lambda(.)phi(.)det(.)     4 161.28  6.68 0.016
## Haz. rate-Pois, lambda(.)phi(.)det(.)         3 162.07  7.47 0.011
##                                           cumltvWt
## Half.-Neg. Bin, lambda(.)phi(.)det(.)         0.46
## Half.-Pois. lambda(.)phi(.)det(.)             0.76
## Exp.-Neg. Bin, lambda(.)phi(.)det(.)          0.89
## Exp.-Pois. lambda(.)phi(.)det(.)              0.97
## Haz. rate-Neg. Bin. lambda(.)phi(.)det(.)     0.99
## Haz. rate-Pois, lambda(.)phi(.)det(.)         1.00
```

Results using halfnormal and Negative Binomial

```
#Backtransformed results of the better model:
backTransform(fm0.hn.NB, type="lambda")
## Backtransformed linear combination(s) of Abundance estimate(s)
##
##  Estimate   SE LinComb (Intercept)
##      1.31 0.37   0.268           1
##
## Transformation: exp
```

Goodness of fitting model (GoF)

Check out the goodness-of-fit of this model

```
fitstats <- function(fm) {
  observed <- getY(fm@data)
  expected <- fitted(fm, na.rm = TRUE)
  resids <- residuals(fm, na.rm = TRUE)
  sse <- sum(resids^2, na.rm = TRUE)
  chisq <- sum((observed - expected)^2 / expected, na.rm = TRUE)
  freeTuke <- sum((sqrt(observed) - sqrt(expected))^2, na.rm = TRUE)
  out <- c(SSE=sse, Chisq=chisq, freemanTukey=freeTuke)
  return(out)
}

(pb <- parboot(fm0.exp.NB, fitstats, nsim=10000, report=1))
## t0 = 48.42029 424.9238 28.23728
## Running in parallel.  Bootstrapped statistics not reported.
##
## Call:
## parboot(object = fm0.exp.NB, statistic = fitstats, nsim = 10000, report = 1)
##
## Parametric Bootstrap Statistics:
##                 t0 mean(t0 - t_B) StdDev(t0 - t_B) Pr(t_B > t0)
## SSE           48.4           1.95            16.01        0.393
## Chisq        424.9          19.28            73.75        0.325
## freemanTukey  28.2           0.93             4.78        0.417
##
```

```
## t_B quantiles:
##                   0% 2.5% 25% 50% 75% 97.5% 100%
## SSE             10.6   22  35  44  55    85  141
## Chisq          234.5  298 354 393 443   584  986
## freemanTukey     9.6   18  24  27  30    37   46
##
## t0 = Original statistic compuated from data
## t_B = Vector of bootstrap samples
```

Histograms of GoF

```
par(mfrow=c(2,2))
par(mar = c(5.00,
            5.00,
            2.00,
            1.00))
hist(pb@t.star[,1], xlab="SSE", col="lightgrey",
  font.lab=2, cex.lab=1, main="")
abline(v=pb@t0[1], lty=2, lwd=2)
par(mar = c(5.00,
            1.00,
            2.00,
            2.00))
hist(pb@t.star[,2], xlab="Chisq", col="lightgrey",
  font.lab=2, cex.lab=1, main="")
abline(v=pb@t0[2], lty=2, lwd=2)
par(mar = c(5.00,
            5.00,
            1.00,
            0.00))
hist(pb@t.star[,3], xlab="freemanTukey", col="lightgrey", breaks=15,
  font.lab=2, cex.lab=1, main="")
abline(v=pb@t0[3], lty=2, lwd=2)
```

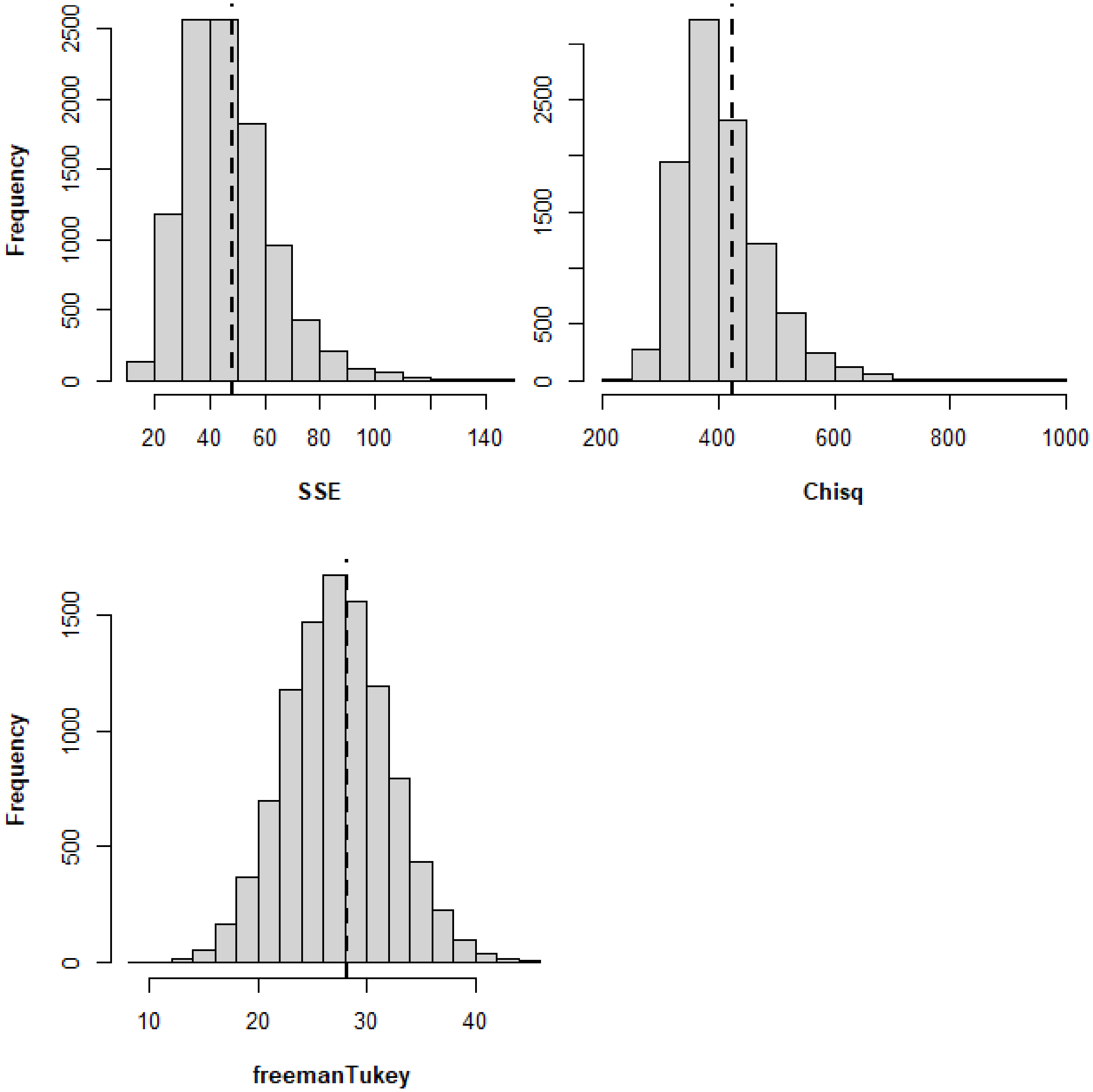

Chisquare test statistic

```
# c-hat as ratio of observed and mean of expected value of Chi2
(c.hat <- pb@t0[2] / mean(pb@t.star[,2]))
##    Chisq
## 1.047529
# Better fit
```
